# LocusPackRat: an R package to support prioritizing candidate genes from large GWAS intervals with standardized evidence aggregation

**DOI:** 10.1093/g3journal/jkag081

**Published:** 2026-03-28

**Authors:** Brian Gural, Todd Kimball, Anh N Luu, Christoph D Rau

**Affiliations:** Department of Genetics, The University of North Carolina at Chapel Hill, Chapel Hill, NC 27599, United States; Computational Medicine Program, The University of North Carolina at Chapel Hill, Chapel Hill, NC 27599, United States; Department of Genetics, The University of North Carolina at Chapel Hill, Chapel Hill, NC 27599, United States; Computational Medicine Program, The University of North Carolina at Chapel Hill, Chapel Hill, NC 27599, United States; Computational Medicine Program, The University of North Carolina at Chapel Hill, Chapel Hill, NC 27599, United States; Department of Pharmacology, The University of North Carolina at Chapel Hill, Chapel Hill, NC 27599, United States; Department of Genetics, The University of North Carolina at Chapel Hill, Chapel Hill, NC 27599, United States; Computational Medicine Program, The University of North Carolina at Chapel Hill, Chapel Hill, NC 27599, United States

**Keywords:** GWAS, candidate gene prioritization, Collaborative Cross, InterMine, prioritization algorithm, R

## Abstract

Genome-wide association studies (GWAS) routinely implicate broad loci that span tens of megabases and contain dozens of genes, making the leap from locus to causal gene challenging, especially in model organism cohorts with reduced mapping resolution. We developed LocusPackRat, an easily extendable R package that assembles standardized “packets” of evidence to accelerate candidate gene prioritization. Each packet merges study-specific information for each gene in a locus such as differential expression between conditions or presence of *cis-*eQTLs with functional/disease annotations pulled from InterMine and Open Targets. Packets are identically structured and easily disseminated to support side-by-side comparison and team review. We demonstrate LocusPackRat’s efficacy on a recent GWAS study of cardiac hypertrophy and failure in the Collaborative Cross. LocusPackRat streamlines the transition from statistical associations to mechanistic hypotheses by providing a systematic, transparent framework for GWAS data integration and is readily adaptable to other genetic reference populations or human cohorts.

## Introduction

Genome-wide association studies (GWAS) and similar approaches (e.g. linkage studies) have led to the identification of many genes which are linked to phenotypes of interest ranging from height (Bicknell et al. 2025) to blood pressure (Padmanabhan and Dominiczak 2021) to cardiovascular disease risk (Rasooly et al. 2023). A frequent bottleneck for these analyses is the transition from identifying loci to candidate genes. This is particularly the case in model organism genetic reference populations (GRPs) where mapping resolution is often lower and, loci routinely span tens of megabases (Peirce et al. 2004; Ghazalpour et al. 2012; Keele et al. 2019; Rau et al. 2020), containing dozens to hundreds of genes.

Several frameworks exist to address this locus-to-gene (L2G) problem in human GWAS. Tools like MAGMA (De Leeuw et al. 2015) and Pascal (Lamparter et al. 2016) provide gene-level associations by aggregating SNP-level *P*-values, offering a statistical basis for differentiating genes within a locus. Broader platforms, such as Open Targets (Ochoa et al. 2021), consolidate genetic and functional evidence to link loci to drug targets. However, these tools are designed around human GWAS summary statistics and large-scale fine-mapping efforts that are either unavailable or poorly suited to model organism GRPs, where sample sizes are smaller, loci are broader, and the relevant annotation databases differ. When these approaches have been applied to model organisms, they have been study-specific and not packaged into reusable tools (Maga et al. 2015; Li et al. 2024). A generalizable, extensible framework for systematically aggregating gene-level evidence across these diverse data types in model organism GRPs is currently lacking. Identifying the best candidate gene from each locus often involves an effort to collate relevant information for each gene followed by locus-wide comparisons to prioritize genes for downstream validation. As part of our work in the Collaborative Cross (CC), a murine GRP, we set out to create an interpretable, easily extendable R package called LocusPackRat that standardizes and streamlines the aggregation of gene-level evidence to accelerate candidate gene nomination and validation. Below, we detail the components of LocusPackRat and apply it to a GWAS from the CC.

## Methods

Although these methods describe resources relevant to our application of LocusPackRat to the CC, equivalent resources exist for other GRPs.

### Desired qualities of high-confidence candidate genes

**Fig. 1.**
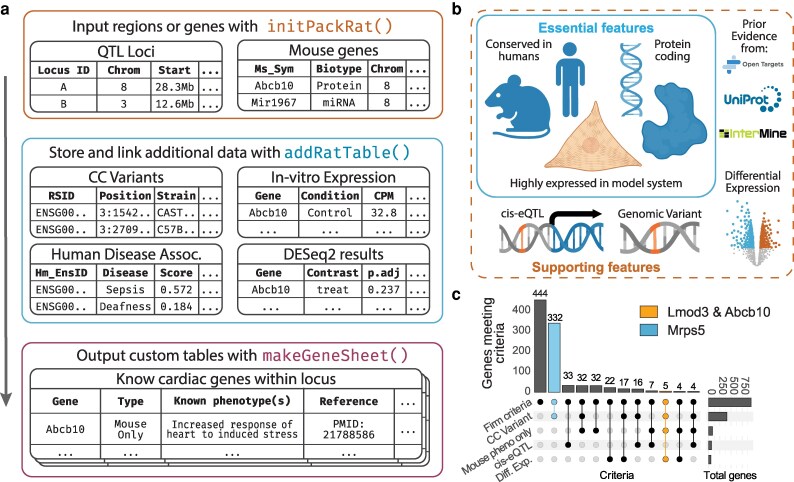
LocusPackRat schema, application, and outcomes of evidence integration. a) Overview of the core LocusPackRat workflow. Users supply QTL intervals or a gene list, initialize a standardized project, attach study-specific or external evidence layers, and generate formatted gene sheets for candidate gene comparison. See Methods for details and additional functions for accessing databases and generating locus zoom plots. b) Essential and supportive criteria used to evaluate candidate genes in our Collaborative Cross heart failure study. c) UpSet plot summarizing evidence overlap across 20 loci associated with heart failure phenotypes in mice. Connected dots indicate included evidence types; “Firm criteria” correspond to the essential features in b). Three genes selected for downstream validation are highlighted: Mrps5 (blue) and Lmod3 and Abcb10 (both orange).

In a locus, there are often tens to hundreds of potential candidate genes to consider, any of which could, in theory, be one of the phenotype-driving genes at the site. Our goal, therefore, is to identify a handful (ideally one) of genes from each locus for further study. The ideal candidate gene would have the following characteristics (Fig. 1b):

Required:

1. Expression in a relevant tissue/cell type
2. Experimentally actionable—protein-coding and with a human ortholog

Supportive (as many as possible):

1. Observed differential expression in response to a relevant stressor
2. Predicted deleterious nonsynonymous variant
3. Detected significant *cis*-eQTL within the locus
4. Recovered prior links in literature or databases to mechanistically relevant traits in either an animal model or humans

### Structure of LocusPackRat

To assist in the identification of genes which meet these criteria, LocusPackRat creates “packets” of information for each locus of a GWAS study, structured identically across all loci to enable direct comparisons, with standardized file naming and organization.

The LocusPackRat workflow proceeds in 3 steps (Fig. 1a). First, initPackRat() takes user-supplied QTL intervals or gene lists and creates a standardized .locusPackRat project directory structure (inputs, cache, supplementary, outputs), harmonizes gene identifiers (gene symbols and Ensembl IDs), and records genome and ortholog metadata. Second, study- or resource-derived evidence is attached to using addRatTable(). Finally, makeGeneSheet() integrates the inputs with user-provided criteria to generate preformatted gene sheets (CSV/Excel) for side-by-side comparison.

In terms of flexibility, initPackRat() requires a specific input format: a data frame with a gene_symbol (gene mode) or chr/start/end columns (region mode). By contrast, addRatTable() accepts any data frame with a linkable column (gene symbol, Ensembl ID, or genomic coordinates), making it straightforward to incorporate arbitrary evidence types. The filtering step in makeGeneSheet() is fully customizable, accepting any valid R expression that evaluates TRUE/FALSE over the merged columns, giving users complete control over how evidence is weighted and combined. All inputs must share the same genome build to ensure correct coordinate matching. For datasets which use a different genome build to the main Pack, we include a liftOver example in the supplementary vignettes (Supplementary File 1) to facilitate coordinate conversion.

Packets include:

- A multi-sheet Excel workbook containing
  - A summary sheet providing basic information regarding the generation of the packet, formatting, and other sheet information
  - A complete sheet containing all genes within each locus that includes all information provided by the user (e.g. expression levels, differential expression, mutations, etc.) along with information pulled from MouseMine (Motenko et al. 2015) and OpenTargets (Ochoa et al. 2021) databases
  - Custom user sheets subset from the complete sheet
  - Sheets from MouseMine and OpenTargets cleanly listing each linked phenotype, pubmedIDs of relevant literature, and the OpenTargets disease score
- LocusZoom (Pruim et al. 2010)-style visualizations displaying the GWAS locus and gene tracks, along with other relevant information, such as haplotype effect trajectories

### Mined information

The mouse genetics community benefits from several large consortia committed to defining the function of every expressed gene in this model organism, with commitments by groups such as the International Mouse Phenotyping Consortium (IMPC) (Groza et al. 2023) and the Knock Out Mouse Project (KOMP) (Austin et al. 2004) to create knockout models of each gene in the genome. The Mouse Genome Informatics (MGI) website has compiled all current phenotype information into a searchable MouseMine database, and we have made this easily queried and integrated into the project through dedicated functions for accessing MGI API via the httr R package (Wickham 2026). Similarly, human disease associations are gathered through the Open Targets GraphQL API (Ochoa et al. 2021).

LocusPackRat returns all mouse and human gene–trait associations from these databases and has a customizable filter for specific traits of interest. We have found it useful to examine this information in 2 passes, first looking for genes explicitly tied to our phenotype of interest to look for known genes that may explain a locus, then a second pass to identify genes with functional evidence of roles in related phenotypes (e.g. high blood pressure vs cardiac hypertrophy) which may represent more novel or less well-studied candidates.

### Sequence-level information

The Wellcome Trust Mouse Genomes Resource (Keane et al. 2011) provides base pair-level resolution for genetic variants in 36 mouse strains. These include the 8 founder lines of the CC, which, when paired with the known haplotype information for each CC strain, allows us to include point mutations in each CC line. Comparable results could be achieved for many other mouse panels, such as the BxD cohort (Peirce et al. 2004) or Hybrid Mouse Diversity Panel (Ghazalpour et al. 2012), each of which have their founder lines present in the sequencing data. Similar datasets exist for other GRPs (Grenier et al. 2015). As interpretation of noncoding mutations can be difficult, we limit ourselves to nonsynonymous, protein-modifying mutations for each protein-coding gene.

### Study-specific data

The data described above are of common interest to any study and can be used to filter candidate genes; however, these lists can be further refined if researchers have additional, study-specific data in the form of phenotypic or transcriptional information, for example, ATAC-seq or CHiP-seq data for open chromatin regions or Hi-C linkages between the tag SNP and individual gene promoters, or simply transcriptional data from RNA-seq or Microarray analyses.

### Molecular QTL integration

Beyond study-specific eQTL data, LocusPackRat provides access to curated pan-study molecular QTL resources through the queryOpenTargetsQTL() function. This function queries the Open Targets Genetics API to retrieve fine-mapped credible sets for expression QTL (eQTL), protein QTL (pQTL), splicing QTL (sQTL), and single-cell QTL (sceqtl, scpqtl) data types drawn from large-scale studies including GTEx, eQTLGen, deCODE, and INTERVAL. Each credible set includes a L2G prediction score, which integrates chromatin interaction, distance, and functional annotation features to assign a probability that a given variant–gene pair is the causal link and can optionally be filtered to keep only high-confidence assignments. GWAS colocalization data, when available, are also returned, providing evidence that a QTL and GWAS signal share a common causal variant. The returned data are automatically attached to the locusPackRat project via addRatTable() for downstream integration and filtering. A worked example showing this workflow is provided in the region_qtl_opentargets vignette (Supplementary File 2).

For model organism GRP-specific QTL data, which are not available through Open Targets, a supplementary vignette shows querying the GeneNetwork2 (genenetwork.org) REST API for BxD and Diversity Outbred eQTL datasets (Supplementary File 3). QTL results retrieved from GeneNetwork2 can be formatted and added as evidence layers via addRatTable(), enabling researchers to combine population-specific molecular QTL data with other evidence in the locusPackRat framework.

Table 1 contains a list of commonly used resources (e.g. GTEx, ChEMBL, Tabula Muris) and how to access them in LocusPackRat.

**Table 1.** Accessing data from other commonly used repositories using LocusPackRat.

Resource	Data types available	LocusPackRat function	Website	Notes
Open Targets	Many	queryOpenTargets()	opentargets.org	Mostly human data
MouseMine	Many	queryMouseMine()	mousemine.org	Includes mouse data from the Jackson Labs
GTEx	Tissue-level expression and eQTL	queryOpenTargetsQTL()	gtexportal.org	Samples in up to 54 tissues from ∼1,000 individuals with expression and genotyping
STRING	Protein	queryOpenTargets() (interactions)	string-db.org	Protein–protein interactions informed either by experimentation or bioinformatics
ClinVar	Gene–disease associations	queryOpenTargets() (diseases)	ncbi.nlm.nih.gov/clinvar	
ChEMBL	Gene–drug interactions	queryOpenTargets() (known_drugs)	ebi.ac.uk/chembl	Updating database of known interactions between drugs and genes
BxD Cohort	Many	See GeneNetwork2 vignette	systems-genetics.org	Data primarily drawn from studies on the BxD Recombinant Inbred Mouse Population
Tabula Sapiens	Single-cell expression	See single-cell vignette	tabula-sapiens.sf.czbiohub.org	Human single-cell expression from 28 organs
Tabula Muris	Single-cell expression	See single-cell vignette	tabula-muris.sf.czbiohub.org	Mouse single-cell expression from 20 organs
NCBI GEO and SRA	Expression	See general vignette	ncbi.nlm.nih.gov/geo ncbi.nlm.nih.gov/sra	Programmatic download possible with E-Utils SRA reads will need to be realigned
NHGRI-EBI GWAS Catalogue	GWAS hits	See general vignette	ebi.ac.uk/gwas	Constantly updated catalog of human GWAS hits R package: gwasrapidd

## Results and discussion

### Collaborative Cross study

We now describe the application of LocusPackRat to our recently reported GWAS on isoproterenol (ISO)-driven cardiac hypertrophy and failure in the CC (Kimball et al. 2026) (Fig. 1c).

Heart failure is characterized by a complex etiology in humans that often manifests as an increase in beta adrenergic signaling and catecholamine-driven overdrive of the heart which leads to failure (Rau et al. 2015). We used the drug isoproterenol, a synthetic beta-adrenergic agonist, to induce heart failure phenotypes in 454 mice from 71 strains of the CC. We then mapped both control and ISO-treated phenotypes to the mm39 genome using the miQTL tool as described (Kimball et al. 2026). We detected 49 genome-wide significant loci, which merged to 20 distinct genomic intervals when overlapping regions and multi-trait loci were considered. These loci averaged 12.8 Mb and held a total of 2,149 genes, which we wanted to prioritize for experimental validation in neonatal rat ventricular cardiomyocytes (NRVMs). Below, we detail the results for all loci, as well as a specific locus on chromosome 3 which shows strong associations with cardiac echocardiographic traits.

### Initial screening

We began by filtering our potential candidate gene pool based on the following criteria: (i) Genes must have obvious human orthologs (74% of all candidate genes), be robustly expressed in the cell type we intended to validate in (NRVMs, 46% of all candidate genes), and, lastly, be protein-coding to prioritize historically more easily manipulatable targets. These criteria gave us the best possibility of being able to query our candidate genes in vitro using *siRNA*-mediated knockdown or adenovirus-associated overexpression studies. This resulted in 950 total genes (44% of the original) that needed further curation.

### Manual prioritization

After producing automated locus packets, the last step of candidate gene prioritization relies on manual curation by the researcher(s). Very few genes (only 1, in the case of the CC study) will possess evidence in all categories, while many genes will show evidence in a few. For example, we observed 407 genes that met our basic criteria and had a nonsynonymous variant in at least one founder strain, but only 50 which were differentially expressed between ISO and Ctrl cohorts (Fig. 1c). A final screen based on researcher domain expertise and with an eye for biological plausibility acts as a powerful final filter for gene validation.

We recommend approaching these prioritization tasks as a team effort. As LocusPackRat provides unified, consistent locus packets, we assigned 2 or 3 lab members (to minimize the effect of one individual's personal biases toward specific pathways or degree of knowledge) to each locus, and each locus was examined for convergent evidence patterns, mechanistic plausibility, tractability for downstream testing in isolated cardiomyocytes, and translational potential for human cardiovascular studies. Results were presented in informal presentations in which evidence, both for and against each gene, was considered before a final prioritized gene candidate list was compiled for each locus.

### Examination of a single CC GWAS locus

**Fig. 2.**
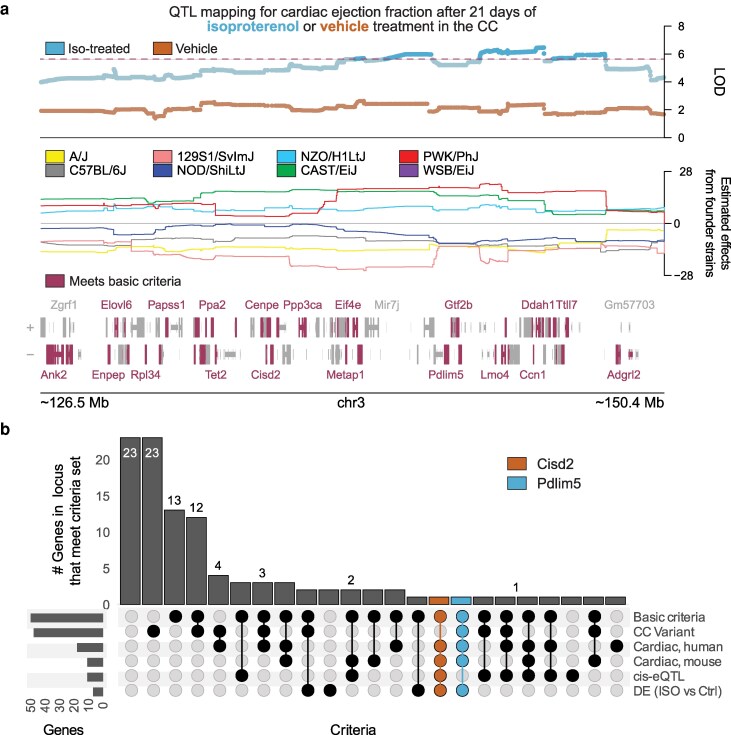
Example application of locusPackRat to a wide locus on mouse chromosome 3. a) Top: Locus zoom plot showing the logarithm of the odds (LOD) for associations between genomic markers in the Collaborative Cross (CC) and measured cardiac ejection fraction after isoproterenol-induced stress (blue) or vehicle (orange). Dashed line shows a genome-wide significance threshold set by permutation testing. Middle: Estimated contributions of CC founder haplotypes to the observed associations. Bottom: Gene tract showing genes within the locus (mm39). Genes that have a known human ortholog, encode protein, and are expressed above 5 cpm in NRVMs were prioritized for manual comparison with locusPackRat. b) UpSet plot showing the outcome of criteria set on all genes within the locus. Compelling candidate genes, Cisd2 and Pdlim5 are colored orange and blue. CC Variant, gene contained a coding variant in the CC; Cardiac, mouse, reported association with cardiac traits in mouse models per Mouse Genome Informatics; Cardiac, human, reported association with human cardiac phenotypes per Open Targets; cis-eQTL, detected as a locally regulated gene in internal eQTL mapping; DE, detected as differentially expressed between treated and control mice in internal analysis. Colored columns indicate columns which contain the 2 genes we chose to highlight from this cluster.

As a concrete example, we apply LocusPackRat to a locus associated with post-ISO cardiac functional parameters on chromosome 3 that spans 19 Mb (131 to 150 Mb) and contains 104 genes (Fig. 2a). Of these, 50 pass our screening step (human ortholog, protein-coding, robustly expressed). Of these 50, 14 have a protein-altering mutation in PWK/PhJ, the most likely driver of this locus (mostly missense mutations but with 2 start-loss variants) while 7 other genes have protein-altering mutations in other strains; 10 have a *cis-*eQTL at this locus; 5 show significant differential expression in response to ISO; and 18 have been previously linked to a cardiovascular-associated trait in MouseMine or OpenTargets. Across all 50 screened genes, only one (the beta-mannosidase enzyme, *Manba*) had both a PWK-mutated protein-altering mutation *and* a cis-eQTL. Among the 5 differentially expressed genes, 4 had either a protein-altering mutation or a *cis*-eQTL; the sole exception was *Ube2d3*, which had neither. Notably, 2 (40%) differentially expressed genes had been previously implicated in mouse or human for another cardiovascular trait, along with 6 (60% of total) *cis-*eQTL genes and 6 genes with nonsynonymous mutations (29% of total) (Fig. 2b). The entire packetmay be found in the supplement in zipped format as output from buildPacket() (Supplementary File 5).

After considering the evidence, our attention settled on 2 genes as candidates for this locus. One is *Cisd2*, a regulator of autophagy that shows a strong *cis-*eQTL at this locus, differential expression between control and ISO conditions, and has been implicated in abnormal skeletal muscle morphology, although its role in cardiac muscle remains to be explored (Chang et al. 2012). The other is *Pdlim5*, a scaffolding protein whose overexpression may lead to dilated cardiomyopathy (Wang et al. 2019) and which has a nonsynonymous mutation in PWK/PhJ in the CC and significantly increased expression after ISO. Next steps would involve examining each of these genes in an in vitro system for further validation.

### Comparison with existing frameworks

Several tools address the L2G problem. MAGMA and Pascal provide gene-level association statistics by aggregating SNP *P*-values using LD structure, offering a statistically principled approach for human GWAS; their output can be incorporated into LocusPackRat as a user-supplied data layer via addRatTable(). The Open Targets Platform consolidates genetic associations, functional genomics, and drug tractability data into a comprehensive portal; LocusPackRat integrates with Open Targets through its queryOpenTargets() and queryOpenTargetsQTL() functions, but additionally allows researchers to layer study-specific data (e.g. transcriptomics, eQTL, mutations) that are not captured in public databases. FUMA (Watanabe et al. 2017) is a web-based platform that performs SNP-to-gene mapping, gene-set enrichment, and tissue expression analysis for human GWAS, but requires uploading summary statistics to an external server and does not support model organism data. By contrast, LocusPackRat is an R-based framework that runs entirely offline, supports both human and model organism cohorts, and is designed to be extended with arbitrary data types. Its key differentiator is the combination of a flexible, additive evidence framework with standardized output that facilitates team-based review, particularly for GRPs where standard LD-based tools are not directly applicable.

### Limitations

We have extensively tested LocusPackRat in the context of heart-related loci from the CC, and its utility in other settings will depend on the availability of equivalent data sources. LocusPackRat was designed to be easily extendable to other GRPs through simple modifications to incorporate panel- and phenotype-specific information, and for maximal efficacy, researchers should have user-supplied sequence-level DNA information and RNA transcriptomes from their study. Equivalent resources to MouseMine exist through intermine.org for many organisms (e.g. FlyMine for Drosophila), and IMPC's and KOMP's phenotypic screens of knockout lines provided further context. Model organisms with fewer of these resources will find LocusPackRat’s automated annotation layers less informative, though the framework should still provide a useful foundation for organizing and comparing gene-level evidence.

Mendelian randomization (MR) methods are increasingly used to move beyond association and infer directional effects between molecular traits (e.g. gene expression) and disease phenotypes using QTL data as instrumental variables. While LocusPackRat does not currently perform MR analyses, users who have conducted MR or colocalization analyses (e.g. with tools such as coloc (Rasooly et al. 2022) or SMR (Jacobs et al. 2020)) could incorporate these results as an added evidence layer via addRatTable(). In the context of model organism GRPs, the small sample sizes and limited availability of large-scale molecular QTL datasets can constrain the power of MR approaches, but as these resources grow, MR-derived evidence is likely to become an increasingly valuable input for candidate gene prioritization.

In some cases, the filtering criteria may eliminate all candidate genes from a locus, leaving no prioritized candidates. When this occurs, we recommend a stepwise approach: First, relax the initial screening criteria (e.g. lower the expression threshold, include noncoding genes, or broaden the ortholog requirement); then, systematically drop supportive criteria one at a time to identify which evidence layer is most restrictive. If no protein-coding gene passes even relaxed filters, this may indicate that the causal signal at the locus operates through a noncoding regulatory mechanism (e.g. a long noncoding RNA, enhancer variant, or structural variant) that is not captured by the current gene-centric evidence layers or that the initial associations are spurious. In such cases, researchers may consider expanding the evidence base by querying additional databases, performing formal colocalization analyses, or examining chromatin accessibility and 3-dimensional genome organization data to identify regulatory elements that may link the locus to a distal target gene.

Further consideration is cell-type specificity. Bulk-tissue transcriptomic and eQTL data, which form a common evidence layer in LocusPackRat workflows, can obscure effects that are specific to cell types. The queryOpenTargetsQTL() function retrieves single-cell eQTL (sceqtl) and single-cell pQTL (scpqtl) data types when available, providing an initial window into cell-type-resolved molecular effects. As single-cell QTL resources continue to expand, these data can be incorporated as additional layers of evidence alongside bulk-tissue results. To further explore cell-type-specific expression of candidate genes, a supplementary vignette (Supplementary File 4) demonstrates using prioritized gene sets as module scores in single-cell RNA-seq data (e.g. Tabula Muris via Seurat’s AddModuleScore function), allowing researchers to assess whether candidates are enriched in disease-relevant cell populations.

### Summary

LocusPackRat is an R package meant to facilitate the often-complicated steps between the identification of a genome-wide significant locus and the identification of the key genes that underlie the locus. By providing a standardized “packet” of information that consolidates information from the original study, species-or-cohort-specific data such as sequencing data, and gene-level information such as known functions and knockout effects, LocusPackRat allows for teams of researchers to work together to prioritize candidate gene lists with everyone having the same data at their disposal, speeding the process of picking candidates.

## Supplementary Material

jkag081_Supplementary_Data

## Data Availability

LocusPackRat is implemented as a modifiable and extendable R package at github.com/RauLabUNC/locusPackRat. The repository includes vignettes demonstrating core workflows (locusPackRat_workflow, Supplementary File 1), Open Targets QTL integration (region_qtl_opentargets, Supplementary File 2), GeneNetwork2 querying for model organism QTL data (genenetwork_qtl, Supplementary File 3), and single-cell RNA-seq integration for cell-type-specific candidate evaluation (single_cell_integration, Supplementary File 4). All data used in the Collaborative Cross case study are available through the supplementary materials. Phenotypes for the CC may be found in the Mendeley Data Repository at doi:10.17632/vyf5x4ygrv.1. LocusPackRat is available at GitHub: github.com/RauLabUNC/locusPackRat. Supplemental material available at G3 online.
