# Supplementary material for: LocusPackRat: an R package to support prioritizing candidate genes from large GWAS intervals with standardized evidence aggregation
Source: G3 (Bethesda). 2026 Mar 28;16(6):jkag081. doi: 10.1093/g3journal/jkag081 (PMC13232493; doi:10.1093/g3journal/jkag081)
Supplement: jkag081_Supplementary_Data [file jkag081_supplementary_data.zip › Supplementary_File_1_G3-2026-406637.html]

locusPackRat: Project-Based Genomic Analysis


# locusPackRat: Project-Based Genomic Analysis

#### Brian Gural, Todd Kimball, Anh Luu, Christoph D. Rau

#### 2026-02-18

- Overview
  - Key Features
  - Data
    Resources
  - Data Sources Used in This
    Vignette
- Installation
- Gene Mode
  Analysis
  - Initialize Project with Gene
    List
  - Add Supplementary Data
    - Tissue Expression Atlas
    - Pathway
      Annotations
    - Add mouseMine and Open
      Targets data
  - Examine
    Package Structure Before Creating Outputs
  - Generate Multi-Sheet Excel
    Workbook
- Region
  Mode Analysis
  - QTL Project
    - Initialize Project with
      Genomic Regions
    - Add Supplementary Data
    - Examine
      Package Structure Before Creating Outputs
    - Generate
      Locus Plot
    - Generate Excel Sheet
  - ATACseq
    Project
    - Initialize
      Project with Genomic Regions - ATACseq
    - Add Chromatin Annotations
    - Examine
      Package Structure Before Creating Output
    - Generate ATACseq
      Region Analysis Workbooks
- Building Shareable Packets
  - Including Supplementary
    CSVs
- Working Across Species
  and Genome Builds
  - Human Gene Analysis
    with Mouse Orthologs
  - Converting Between Genome
    Coordinates
- Working with Projects
  - List
    Available Tables
  - Remove a
    Table
  - Project Configuration
  - Output Options
    - CSV Format
    - Filtered
      Output
    - Selective Table Inclusion
- Converting Genome Builds
- Session
  Information

# Overview

`locusPackRat` is a coordinate-based genomic analysis
package for R that provides a project-based workflow with persistent
data storage. The package enables users to integrate multiple data types
and generate professional multi-sheet Excel workbooks for genomic
analyses.

`locusPackRat` functions can be grouped into the following
categories:

- **Project initialization functions:** Create and
  configure analysis projects for genes or genomic regions
- **Data integration functions:** Add supplementary data
  tables that link to genes or regions
- **Output generation functions:** Export integrated data
  as CSV or multi-sheet Excel workbooks

## Key Features

- **Persistent project storage:** Load data once, use
  many times
- **Flexible data integration:** Link any supplementary
  data by gene symbol, Ensembl ID, or coordinates
- **Genome-wide annotations:** Built-in coordinate files
  for ~60,000 mouse and ~30,000 human genes
- **Cross-species support:** Automatic orthology mapping
  between human and mouse
- **Professional outputs:** Multi-sheet Excel workbooks
  with conditional formatting

## Data Resources

The package includes complete genome-wide coordinate files generated
from Ensembl Biomart:

- `mouse_coords_mm39.csv`: ~60,000 mouse genes
  (GRCm39)
- `mouse_coords_mm10.csv`: ~38,000 mouse genes
  (GRCm10)
- `human_coords_hg38.csv`: ~30,000 human genes
  (GRCh38)
- `human_coords_hg19.csv`: ~24,000 human genes
  (GRCh19)

## Data Sources Used in This Vignette

Throughout this vignette, we draw on the following data types:

- **Gene coordinates and orthology**: Built-in Ensembl
  Biomart coordinate files and human-mouse orthology tables
- **MouseMine phenotype annotations**: Gene-phenotype
  associations from the Mouse Genome Informatics (MGI) database, queried
  via `queryMouseMine()`
- **Open Targets disease associations**: Human
  gene-disease links, genetic constraints (gnomAD), and drug tractability
  data, queried via `queryOpenTargets()`
- **Study-specific data**: User-supplied tables (e.g.,
  differential expression, eQTL, mutations) added via
  `addRatTable()`

For molecular QTL data from Open Targets (eQTL, pQTL, sQTL), see the
`region_qtl_opentargets` vignette. For model organism QTL
data from GeneNetwork2, see the `genenetwork_qtl` vignette.
For cell-type-specific evaluation of candidate genes using single-cell
RNA-seq, see the `single_cell_integration` vignette.

# Installation

```
# Install from GitHub
#devtools::install_github("RauLabUNC/locusPackRat")

#Or, install locally
#devtools::install_local("Path/To/File/locusPackRat.zip")

# Load required libraries
library(locusPackRat)
library(data.table)
library(jsonlite)
```

# Gene Mode Analysis

Gene mode is designed for analyzing gene lists from RNA-seq or other
gene-centric studies. In this mode, you provide a data.frame with a
`gene_symbol` column and any additional columns of per-gene
data (e.g., log2 fold changes, p-values). `initPackRat()`
maps these gene symbols to genomic coordinates and creates the project
directory structure. See below for Region Mode, designed for analysis of
a chromosome region.

## Initialize Project with Gene List

The first step is to call `initPackRat()` with your gene
data. The `mode = "gene"` argument tells the function to
expect a gene list rather than genomic intervals. The
`species` and `genome` arguments determine which
built-in coordinate file is used for mapping.
**Important**: all data added to the project later must use
the same genome build.

```
# Prepare example RNA-seq differential expression results
set.seed(27)
rna_genes <- data.table(
  gene_symbol = c(
    c("Myc", "Tp53", "Egfr", "Vegfa", "Il6", "Tnf", "Apoe", "Lep", "Ins2", "Bdnf"),
    sample(fread(system.file("extdata", "mouse_coords_mm39.csv",
                             package = "locusPackRat"))$gene_symbol, 140)
  ),
  log2FC = round(rnorm(150, mean = 0, sd = 2), 2),
  padj = 10^(-runif(150, 0.5, 10)),
  baseMean = round(10^runif(150, 1, 5), 1),
  direction = ifelse(rnorm(150) > 0, "up", "down")
)

# Initialize project - automatically adds genomic coordinates
initPackRat(
  data = rna_genes,
  mode = "gene",
  species = "mouse",
  genome = "mm39",
  project_dir = "rnaseq_analysis",
  force = TRUE
)
#> Initializing locusPackRat project...
#> Note: All data added to this project must use the mm39 genome build. Mixing genome builds will cause incorrect coordinate matching. Use rtracklayer::liftOver() to convert coordinates if needed.
#> Processing gene list...
#>   Matched 149/150 genes to coordinates (1 not found: Tp53)
#> Generating orthology information...
#> Saved gene data to rnaseq_analysis/.locusPackRat/input/genes.csv
#> Saved orthology data to rnaseq_analysis/.locusPackRat/input/orthology.csv
#> Created config file: rnaseq_analysis/.locusPackRat/config.json
#> 
#> locusPackRat project initialized successfully!
#> Mode: gene | Species: mouse | Genome: mm39
#> Processed 150 genes
```

## Add Supplementary Data

Supplementary data tables are added to the project using
`addRatTable()`. Each table must contain a column that can be
linked to the gene list — either `gene_symbol`,
`ensembl_gene_id`, or genomic coordinates (`chr`,
`start`, `end`). The `link_type`
parameter specifies how the data connects to the project genes, and
`link_by` identifies the linking column(s).

### Tissue Expression Atlas

Tissue expression data helps identify whether candidate genes are
expressed in disease-relevant tissues. Here we simulate TPM values
across six tissues and calculate tissue specificity metrics.

```
# Load processed genes with coordinates
processed_genes <- fread("rnaseq_analysis/.locusPackRat/input/genes.csv")

# Add tissue expression data (TPM values)
tissue_expression <- data.table(
  gene_symbol = processed_genes$gene_symbol,
  brain = round(abs(rnorm(nrow(processed_genes), 50, 30)), 1),
  liver = round(abs(rnorm(nrow(processed_genes), 40, 25)), 1),
  heart = round(abs(rnorm(nrow(processed_genes), 45, 28)), 1),
  kidney = round(abs(rnorm(nrow(processed_genes), 35, 20)), 1),
  lung = round(abs(rnorm(nrow(processed_genes), 42, 24)), 1),
  muscle = round(abs(rnorm(nrow(processed_genes), 38, 22)), 1)
)

# Calculate tissue specificity
tissue_expression[, max_tissue := names(.SD)[max.col(.SD)],
                 .SDcols = c("brain", "liver", "heart", "kidney", "lung", "muscle")]
tissue_expression[, tissue_specific := apply(.SD, 1, function(x) max(x)/mean(x) > 2),
                 .SDcols = c("brain", "liver", "heart", "kidney", "lung", "muscle")]

addRatTable(
  data = tissue_expression,
  table_name = "tissue_expression",
  abbreviation = "te",
  link_type = "gene",
  link_by = "gene_symbol",
  project_dir = "rnaseq_analysis"
)
```

### Pathway Annotations

Pathway membership data provides functional context for candidate
genes. Note that `addRatTable()` accepts any data.frame — the
only requirement is that it contain a column matching
`link_by` that can be joined to the project gene list.

```
# Add pathway membership data
pathways <- c("Cell cycle", "Apoptosis", "Immune response", "Metabolism",
              "Signal transduction", "DNA repair", "Protein synthesis")

pathway_data <- data.table(
  gene_symbol = sample(processed_genes$gene_symbol, 80, replace = TRUE),
  pathway = sample(pathways, 80, replace = TRUE),
  evidence = sample(c("experimental", "computational", "literature"), 80, replace = TRUE)
)

addRatTable(
  data = unique(pathway_data),
  table_name = "pathway_annotations",
 # abbreviation = "pa",
  link_type = "gene",
  link_by = "gene_symbol",
  project_dir = "rnaseq_analysis"
)
```

### Add mouseMine and Open Targets data

`queryMouseMine()` retrieves gene-phenotype associations
from the Mouse Genome Informatics database, and
`queryOpenTargets()` retrieves human disease associations,
genetic constraint scores (gnomAD), and drug tractability data. Both
functions query their respective APIs and automatically store results as
supplementary tables in the project. For mouse projects, genes are
mapped to human orthologs before querying Open Targets.

```
queryMouseMine(project_dir = "rnaseq_analysis")
queryOpenTargets(project_dir = "rnaseq_analysis")
```

## Examine Package Structure Before Creating Outputs

```
listPackRatTables(project_dir = "rnaseq_analysis", full_info = TRUE)
#> Found 6 supplementary table(s):
#>   - mouse_phenotypes: 3592 rows with 7 cols, linked by 'gene_symbol'
#>   - ot_constraints: 9 rows with 10 cols, linked by 'gene_symbol'
#>   - ot_diseases: 389 rows with 6 cols, linked by 'gene_symbol'
#>   - ot_tractability: 168 rows with 6 cols, linked by 'gene_symbol'
#>   - pathway_annotations: 79 rows with 4 cols, linked by 'gene_symbol'
#>   - tissue_expression: 150 rows with 10 cols, linked by 'gene_symbol'
#> Printing full column names for 6 supplementary table(s)
#> Columns in tissue_expression:
#> gene_symbol ; ensembl_id ; brain ; liver ; heart ; kidney ; lung ; muscle ; max_tissue ; tissue_specific
#> Columns in pathway_annotations:
#> gene_symbol ; ensembl_id ; pathway ; evidence
#> Columns in mouse_phenotypes:
#> gene_symbol ; ensembl_id ; mgi_id ; mp_id ; phenotype ; pubmed_id ; description
#> Columns in ot_diseases:
#> gene_symbol ; ensembl_id ; human_ensembl_id ; disease_id ; disease_name ; score
#> Columns in ot_constraints:
#> gene_symbol ; ensembl_id ; human_ensembl_id ; constraintType ; exp ; obs ; score ; oe ; oeLower ; oeUpper
#> Columns in ot_tractability:
#> gene_symbol ; ensembl_id ; human_ensembl_id ; label ; modality ; value
#> Completed
#>             table_name table_abbr link_type     link_by n_rows n_cols
#>                 <char>     <char>    <char>      <char>  <int>  <int>
#> 1:    mouse_phenotypes         mm      gene gene_symbol   3592      7
#> 2:      ot_constraints        otc      gene gene_symbol      9     10
#> 3:         ot_diseases        otd      gene gene_symbol    389      6
#> 4:     ot_tractability        ott      gene gene_symbol    168      6
#> 5: pathway_annotations       <NA>      gene gene_symbol     79      4
#> 6:   tissue_expression         te      gene gene_symbol    150     10
#>    date_added
#>        <char>
#> 1: 2026-02-18
#> 2: 2026-02-18
#> 3: 2026-02-18
#> 4: 2026-02-18
#> 5: 2026-02-18
#> 6: 2026-02-18
```

## Generate Multi-Sheet Excel Workbook

`makeGeneSheet()` merges all supplementary tables with the
gene list and generates output workbooks. The
`split_criteria` parameter accepts a named list of R
expressions that define filtered subsets — each becomes a separate sheet
in the Excel workbook. This is the key customization point: you can
combine any columns from any attached table in your filter
expressions.

Additional sheets can be created from any boolean (True/False)
argument. Boolean statements in R can be strung together with &
(and) or | (or). We recommend examining the package structure above with
full\_info = TRUE to identify column names

Note: beware of capitalization. For whatever reason mouseMine does
not capitalize any terms other than gene names, while OpenTargets is
somewhat inconsistent. If needed, more complex arguments e.g. (‘Cardiac’
| ‘cardiac’) & padj <.001 may be used.

Note 2: If two supplemental tables share a column name, the code will
append the table’s abbreviation to the column name: (e.g. if the table
abbreviation is ‘ab’ and the column is ‘value’, it will become
‘ab\_value’). It is also possible to set the prefix\_mode argument below
to “abbreviated”, in which case all columns from tables with an
abbreviation will have it appended by default.

```
# Create Excel workbook with multiple filtered views
makeGeneSheet(
  format = "excel",
  output_file = "rnaseq_complete_analysis.xlsx",
  split_by = "criteria",
  prefix_mode = "collision",
    
  split_criteria = list(
    "All_DEGs" = "TRUE",
    "Upregulated" = "direction == 'up'",
    "Downregulated" = "direction == 'down'",
    "High_Confidence" = "padj < 0.001 & abs(log2FC) > 2",
    "Brain_Specific" = "max_tissue == 'brain' & tissue_specific == TRUE",
    "Immune_Genes" = "pathway == 'Immune response'",
    "mouseMine_Immune" = "grepl('immune',phenotype)"
  ),
  highlight_genes = c("Myc", "Tp53", "Egfr", "Il6", "Tnf"),
  include_supplementary = TRUE,
  project_dir = "rnaseq_analysis"
)
```

# Region Mode Analysis

Region mode is designed for analyzing genomic intervals from
ATAC-seq, ChIP-seq, or QTL mapping. In this mode, you provide a
data.frame with `chr`, `start`, and
`end` columns defining the genomic intervals.
`initPackRat()` identifies all genes overlapping these
regions using the built-in coordinate files and creates the project
structure.

We include two versions of Region Mode Analysis: The first for QTL
mapping, the second for ATAC-seq data.

## QTL Project

### Initialize Project with Genomic Regions

```
#LocusPackRat includes an example QTL output with a single significant locus on chromsome 3.
#Here we examine two regions, one significant and the other one insignificant

QTL_peaks <- data.table(
  chr = as.character(c(3,6)),
  start=c(126967000,50000000),
  end=c(149860000,60000000),
  peak_id=c("Significant_1","Insignificant_1"),
  max_lod=c(5.64, 3.21)
)

initPackRat(
  data = QTL_peaks,
  mode = "region",
  species = "mouse",
  genome = "mm39",
  project_dir = "qtl_analysis",
  keep_pseudo=F,
  force = TRUE
)
#> Initializing locusPackRat project...
#> Note: All data added to this project must use the mm39 genome build. Mixing genome builds will cause incorrect coordinate matching. Use rtracklayer::liftOver() to convert coordinates if needed.
#> Processing region list...
#> Generating orthology information...
#> Saved region data to qtl_analysis/.locusPackRat/input/regions.csv
#> Saved orthology data to qtl_analysis/.locusPackRat/input/orthology.csv
#> Created config file: qtl_analysis/.locusPackRat/config.json
#> 
#> locusPackRat project initialized successfully!
#> Mode: region | Species: mouse | Genome: mm39
#> Processed 2 regions
```

### Add Supplementary Data

In region mode, supplementary data is linked to the project using
`addRatTable()` with `link_type = "region"` and
`link_by = "chr,start,end"`. Region-level linking assigns
each row of the supplementary table to every gene whose coordinates
overlap the row’s genomic interval. This is appropriate for positional
data such as QTL scan results, variant calls, or ChIP-seq peaks. For
gene-level data (e.g., summary statistics per gene), use
`link_type = "gene"` with
`link_by = "gene_symbol"` as shown in the gene mode section
above.

#### Add QTL Data and CC Founder Information

```
QTL_file <- system.file("extdata", "sample_scan.csv", package = "locusPackRat")
QTL_Data <- fread(QTL_file)

QTL_Data <- data.table(
  chr = QTL_Data$chr,
  start = QTL_Data$pos,
  end = QTL_Data$pos,
  lod = QTL_Data$lod,
  marker = QTL_Data$marker
)

addRatTable(
  data = QTL_Data,
  table_name = "QTL_results",
  abbreviation = "qr",
  link_type = "region",
  link_by = "chr,start,end",
  project_dir = "qtl_analysis"
)

founder_file <- system.file("extdata", "sample_founders.csv", package = "locusPackRat")
founder_Data <- fread(founder_file)

addRatTable(
  data = founder_Data,
  table_name = "founder_info",
  abbreviation = "fi",
  link_type = "region",
  link_by = "chr,start,end",
  project_dir = "qtl_analysis"
)
```

#### Add Mutation Information

Non-synonymous mutation information is provided for these two loci
and drawn from the Wellcome Trust Mouse Genomes Resource

```
SNP_file <- system.file("extdata", "sample_NS_SNP.csv", package = "locusPackRat")
SNP_Data <- fread(SNP_file)
SNP_Data$start <- SNP_Data$pos
SNP_Data$end <- SNP_Data$pos

addRatTable(
  data = SNP_Data,
  table_name = "NS_SNP_information",
  abbreviation = "NSi",
  link_type = "region",
  link_by = "chr,start,end",
  project_dir = "qtl_analysis"
)
```

#### Add mouseMine and Open Targets data

`queryMouseMine()` queries the MouseMine API (hosted by
MGI) and returns all gene-phenotype associations for genes in the
project, stored as a supplementary table named `mouseMine`.
`queryOpenTargets()` queries the Open Targets GraphQL API for
human disease associations, gnomAD genetic constraint scores, and drug
tractability assessments; for mouse projects, genes are automatically
mapped to human orthologs before querying. Results are stored as
separate supplementary tables (`openTargets_diseases`,
`openTargets_constraints`,
`openTargets_tractability`). For molecular QTL data (eQTL,
pQTL, single-cell QTL), see the `region_qtl_opentargets`
vignette.

```
queryMouseMine(project_dir = "qtl_analysis")
queryOpenTargets(project_dir = "qtl_analysis")
```

### Examine Package Structure Before Creating Outputs

```
listPackRatTables(project_dir = "qtl_analysis", full_info = TRUE)
#> Found 7 supplementary table(s):
#>   - founder_info: 76586 rows with 13 cols, linked by 'chr,start,end'
#>   - mouse_phenotypes: 3259 rows with 6 cols, linked by 'gene_symbol'
#>   - NS_SNP_information: 87 rows with 10 cols, linked by 'chr,start,end'
#>   - ot_constraints: 69 rows with 9 cols, linked by 'gene_symbol'
#>   - ot_diseases: 3637 rows with 5 cols, linked by 'gene_symbol'
#>   - ot_tractability: 644 rows with 5 cols, linked by 'gene_symbol'
#>   - QTL_results: 76586 rows with 5 cols, linked by 'chr,start,end'
#> Printing full column names for 7 supplementary table(s)
#> Columns in QTL_results:
#> chr ; start ; end ; lod ; marker
#> Columns in founder_info:
#> A ; B ; C ; D ; E ; F ; G ; H ; marker ; pos ; chr ; start ; end
#> Columns in NS_SNP_information:
#> marker_id ; chr ; pos ; Major ; Minor ; rsID ; MAF ; SNPStrains ; start ; end
#> Columns in mouse_phenotypes:
#> gene_symbol ; mgi_id ; mp_id ; phenotype ; pubmed_id ; description
#> Columns in ot_diseases:
#> gene_symbol ; human_ensembl_id ; disease_id ; disease_name ; score
#> Columns in ot_constraints:
#> gene_symbol ; human_ensembl_id ; constraintType ; exp ; obs ; score ; oe ; oeLower ; oeUpper
#> Columns in ot_tractability:
#> gene_symbol ; human_ensembl_id ; label ; modality ; value
#> Completed
#>            table_name table_abbr link_type       link_by n_rows n_cols
#>                <char>     <char>    <char>        <char>  <int>  <int>
#> 1:       founder_info         fi    region chr,start,end  76586     13
#> 2:   mouse_phenotypes         mm      gene   gene_symbol   3259      6
#> 3: NS_SNP_information        NSi    region chr,start,end     87     10
#> 4:     ot_constraints        otc      gene   gene_symbol     69      9
#> 5:        ot_diseases        otd      gene   gene_symbol   3637      5
#> 6:    ot_tractability        ott      gene   gene_symbol    644      5
#> 7:        QTL_results         qr    region chr,start,end  76586      5
#>    date_added
#>        <char>
#> 1: 2026-02-18
#> 2: 2026-02-18
#> 3: 2026-02-18
#> 4: 2026-02-18
#> 5: 2026-02-18
#> 6: 2026-02-18
#> 7: 2026-02-18
```

### Generate Locus Plot

```
generateLocusZoomPlot(
     region_id="region_1",
     project_dir="qtl_analysis",
     scan_table="QTL_results",
     signal_table="founder_info",
     width=10,
     height=6,
     threshold=4,
     layout_ratios = c(manhattan = 0.35, signal = 0.40, genes = 0.25))
```

### Generate Excel Sheet

Additional sheets can be created from any boolean (True/False)
argument. Boolean statements in R can be strung together with &
(and) or | (or). We recommend examining the package structure above with
full\_info = TRUE to identify column names.

For Region-based packages, one likely does not wish to include all
region-based information in the output (for example, the LoD scores from
the genome scan itself). The exclude\_tables argument will allow you to
exclude these from your output

Note: beware of capitalization. For some reason mouseMine does not
capitalize any terms other than gene names, while OpenTargets is
somewhat inconsistent. If needed, more complex arguments
e.g. grepl(“[c|C]ardiac”,mm\_phenotype) & padj <.001 may be
used.

Note 2: If two supplemental tables share a column name, the code will
append the table’s abbreviation to the column name: (e.g. if the table
abbreviation is ‘ab’ and the column is ‘value’, it will become
‘ab\_value’). It is also possible to set the prefix\_mode argument below
to “abbreviated”, in which case all columns from tables with an
abbreviation will have it appended by default.

```
# Peak-centric analysis workbook
makeGeneSheet(
  format = "excel",
  output_file = "qtl_analysis.xlsx",
  prefix_mode = "abbreviated",
  split_by = "criteria",
  split_criteria = list(
    "All_Results" = "TRUE",
    "Chr 3 Peak" = "peak_id == 'Significant_1'",
    "Mouse_Phenotype" = "grepl('[c|C]ardiac',mm_phenotype) | grepl('[h|H]eart',mm_phenotype) ",
    "Sig OTC" = "(otc_score>1 | otc_score< -1) & peak_id == 'Significant_1'"
  ),
  include_supplementary =  TRUE,
  exclude_tables=c("founder_info","QTL_results"),
  project_dir = "qtl_analysis"
)
```

## ATACseq Project

### Initialize Project with Genomic Regions - ATACseq

```
# Prepare example ATAC-seq peaks
set.seed(123)
chromosomes <- c(as.character(1:19), "X", "Y")

atac_peaks <- data.table(
  chr = sample(chromosomes, 200, replace = TRUE,
               prob = c(rep(0.06, 19), 0.07, 0.07)),
  start = sample(1:150000000, 200),
  peak_id = paste0("peak_", 1:200),
  fold_enrichment = round(runif(200, 2, 50), 2),
  qvalue = 10^(-runif(200, 1, 20))
)

# Calculate end positions (typical ATAC peak width: 200-1000bp)
atac_peaks[, end := start + sample(200:1000, .N, replace = TRUE)]
atac_peaks[, summit := start + round((end - start) / 2)]
atac_peaks[, peak_type := sample(c("promoter", "enhancer", "intergenic"),
                                 .N, replace = TRUE,
                                 prob = c(0.3, 0.4, 0.3))]

# Initialize region-based project
initPackRat(
  data = atac_peaks,
  mode = "region",
  species = "mouse",
  genome = "mm39",
  project_dir = "atacseq_analysis",
  force = TRUE
)
#> Initializing locusPackRat project...
#> Note: All data added to this project must use the mm39 genome build. Mixing genome builds will cause incorrect coordinate matching. Use rtracklayer::liftOver() to convert coordinates if needed.
#> Processing region list...
#> Generating orthology information...
#> Saved region data to atacseq_analysis/.locusPackRat/input/regions.csv
#> Saved orthology data to atacseq_analysis/.locusPackRat/input/orthology.csv
#> Created config file: atacseq_analysis/.locusPackRat/config.json
#> 
#> locusPackRat project initialized successfully!
#> Mode: region | Species: mouse | Genome: mm39
#> Processed 200 regions
```

### Add Chromatin Annotations

#### Chromatin States

```
# Load processed regions
regions <- fread("atacseq_analysis/.locusPackRat/input/regions.csv")

# Add chromatin state annotations
chromatin_states <- data.table(
  chr = regions$chr,
  start = regions$start,
  end = regions$end,
  state = sample(c("Active_Promoter", "Strong_Enhancer", "Weak_Enhancer",
                  "Poised_Promoter", "Repressed", "Heterochromatin", "Transcribed"),
                size = nrow(regions), replace = TRUE,
                prob = c(0.15, 0.2, 0.15, 0.1, 0.1, 0.1, 0.2)),
  cell_type = sample(c("ES_cells", "Neural", "Hepatocyte", "T_cells"),
                    size = nrow(regions), replace = TRUE)
)

addRatTable(
  data = chromatin_states,
  table_name = "chromatin_states",
  abbreviation = "cs",
  link_type = "region",
  link_by = "chr,start,end",
  project_dir = "atacseq_analysis"
)
```

#### Transcription Factor Motifs

```
# Add TF binding motif data
tf_list <- c("CTCF", "NFKB", "AP1", "ETS1", "GATA1", "SOX2", "OCT4", "NANOG",
            "MYC", "MAX", "STAT3", "CREB", "SP1", "YY1", "REST")

# Create random starts
random_starts <- sample(regions$start, 120, replace = TRUE)

motif_data <- data.table(
  chr = sample(regions$chr, 120, replace = TRUE),
  start = random_starts,
  end = random_starts + sample(50:200, 120, replace = TRUE),
  tf_name = sample(tf_list, 120, replace = TRUE),
  motif_score = round(runif(120, 6, 15), 2),
  p_value = 10^(-runif(120, 2, 8))
)

# Add additional motifs for some peaks
additional_motifs <- motif_data[sample(1:nrow(motif_data), 30)]
additional_motifs[, tf_name := sample(tf_list, .N, replace = TRUE)]
motif_data <- rbind(motif_data, additional_motifs)

addRatTable(
  data = motif_data,
  table_name = "tf_motifs",
  abbreviation = "tf_motifs",
  link_type = "region",
  link_by = "chr,start,end",
  project_dir = "atacseq_analysis"
)
```

#### Differential Accessibility

```
# Add differential accessibility between conditions
diff_access <- data.table(
  chr = regions$chr,
  start = regions$start,
  end = regions$end,
  condition_A_signal = round(abs(rnorm(nrow(regions), 100, 50)), 1),
  condition_B_signal = round(abs(rnorm(nrow(regions), 100, 50)), 1),
  log2FC = round(rnorm(nrow(regions), 0, 1.5), 2),
  padj = 10^(-runif(nrow(regions), 0, 8)),
  higher_in = ifelse(rnorm(nrow(regions)) > 0, "condition_B", "condition_A"),
  significant = runif(nrow(regions)) < 0.3
)

addRatTable(
  data = diff_access,
  table_name = "differential_accessibility",
  abbreviation = "da",
  link_type = "region",
  link_by = "chr,start,end",
  project_dir = "atacseq_analysis"
)
```

#### Add mouseMine and Open Targets data

As in the QTL example above, `queryMouseMine()` and
`queryOpenTargets()` retrieve gene-phenotype and gene-disease
annotations for all genes overlapping the ATAC-seq peak regions. These
annotations are stored as supplementary tables and merged with the
peak-level data during output generation.

```
queryMouseMine(project_dir = "atacseq_analysis")
queryOpenTargets(project_dir = "atacseq_analysis")
```

### Examine Package Structure Before Creating Output

```
listPackRatTables(project_dir = "atacseq_analysis", full_info = TRUE)
#> Found 7 supplementary table(s):
#>   - chromatin_states: 200 rows with 5 cols, linked by 'chr,start,end'
#>   - differential_accessibility: 200 rows with 9 cols, linked by 'chr,start,end'
#>   - mouse_phenotypes: 1404 rows with 6 cols, linked by 'gene_symbol'
#>   - ot_constraints: 45 rows with 9 cols, linked by 'gene_symbol'
#>   - ot_diseases: 3192 rows with 5 cols, linked by 'gene_symbol'
#>   - ot_tractability: 420 rows with 5 cols, linked by 'gene_symbol'
#>   - tf_motifs: 150 rows with 6 cols, linked by 'chr,start,end'
#> Printing full column names for 7 supplementary table(s)
#> Columns in chromatin_states:
#> chr ; start ; end ; state ; cell_type
#> Columns in tf_motifs:
#> chr ; start ; end ; tf_name ; motif_score ; p_value
#> Columns in differential_accessibility:
#> chr ; start ; end ; condition_A_signal ; condition_B_signal ; log2FC ; padj ; higher_in ; significant
#> Columns in mouse_phenotypes:
#> gene_symbol ; mgi_id ; mp_id ; phenotype ; pubmed_id ; description
#> Columns in ot_diseases:
#> gene_symbol ; human_ensembl_id ; disease_id ; disease_name ; score
#> Columns in ot_constraints:
#> gene_symbol ; human_ensembl_id ; constraintType ; exp ; obs ; score ; oe ; oeLower ; oeUpper
#> Columns in ot_tractability:
#> gene_symbol ; human_ensembl_id ; label ; modality ; value
#> Completed
#>                    table_name table_abbr link_type       link_by n_rows n_cols
#>                        <char>     <char>    <char>        <char>  <int>  <int>
#> 1:           chromatin_states         cs    region chr,start,end    200      5
#> 2: differential_accessibility         da    region chr,start,end    200      9
#> 3:           mouse_phenotypes         mm      gene   gene_symbol   1404      6
#> 4:             ot_constraints        otc      gene   gene_symbol     45      9
#> 5:                ot_diseases        otd      gene   gene_symbol   3192      5
#> 6:            ot_tractability        ott      gene   gene_symbol    420      5
#> 7:                  tf_motifs  tf_motifs    region chr,start,end    150      6
#>    date_added
#>        <char>
#> 1: 2026-02-18
#> 2: 2026-02-18
#> 3: 2026-02-18
#> 4: 2026-02-18
#> 5: 2026-02-18
#> 6: 2026-02-18
#> 7: 2026-02-18
```

### Generate ATACseq Region Analysis Workbooks

Additional sheets can be created from any boolean (True/False)
argument. Boolean statements in R can be strung together with &
(and) or | (or). We recommend examining the package structure above with
full\_info = TRUE to identify column names.

For Region-based packages, one likely does not wish to include all
region-based information in the output (for example, the LoD scores from
the genome scan itself). See below how to use include\_supplementary to
include only the tables you desire

Note: beware of capitalization. For some reason mouseMine does not
capitalize any terms other than gene names, while OpenTargets is
somewhat inconsistent. If needed, more complex arguments
e.g. grepl(“[c|C]ardiac”,mm\_phenotype) & padj <.001 may be
used.

Note 2: If two supplemental tables share a column name, the code will
append the table’s abbreviation to the column name: (e.g. if the table
abbreviation is ‘ab’ and the column is ‘value’, it will become
‘ab\_value’). It is also possible to set the prefix\_mode argument below
to “abbreviated”, in which case all columns from tables with an
abbreviation will have it appended by default.

```
# Peak-centric analysis workbook
makeGeneSheet(
  format = "excel",
  output_file = "atacseq_peak_analysis.xlsx",
  split_by = "criteria",
  split_criteria = list(
    "All_Peaks" = "TRUE",
    "High_Enrichment" = "fold_enrichment > 20",
    "Promoter_Peaks" = "peak_type == 'promoter'",
    "Enhancer_Peaks" = "peak_type == 'enhancer'",
    "Active_Chromatin" = "state %in% c('Active_Promoter', 'Strong_Enhancer')",
    "Differential" = "significant == TRUE & abs(log2FC) > 1"
  ),
  include_supplementary = TRUE,
  project_dir = "atacseq_analysis"
)

# TF-centric analysis workbook
makeGeneSheet(
  format = "excel",
  output_file = "atacseq_tf_binding.xlsx",
  split_by = "criteria",
  split_criteria = list(
    "CTCF_Binding" = "tf_name == 'CTCF'",
    "Pioneer_TFs" = "tf_name %in% c('SOX2', 'OCT4', 'NANOG')",
    "Immune_TFs" = "tf_name %in% c('NFKB', 'AP1', 'STAT3')",
    "High_Motif_Score" = "motif_score > 12"
  ),
  include_supplementary = c("tf_motifs", "chromatin_states"),
  project_dir = "atacseq_analysis"
)
```

# Building Shareable Packets

Once an analysis is complete, `buildPacket()` bundles
everything in `.locusPackRat/output/` into a single zip
archive with an auto-generated `README.md` summarizing the
project metadata, supplementary data sources, and included files. This
makes it easy to share results with collaborators who may not have R
installed.

```
# Package the QTL analysis for sharing
buildPacket(project_dir = "qtl_analysis", overwrite = TRUE)
#> Packet created: /proj/raulab/users/brian/packrat/vignettes/qtl_analysis/qtl_analysis_packet.zip (595.7 KB)
#> Contains 3 file(s)
```

The zip contains every file from the project’s output directory
(Excel workbooks, locus zoom plots, CSVs) plus a README with project
metadata drawn from `config.json` and
`listPackRatTables()`.

You can also customize the output location and filename:

```
# Write to a specific directory with a custom name
buildPacket(
  project_dir = "rnaseq_analysis",
  output_path = "~/shared_results",
  filename = "rnaseq_v2_packet.zip",
  overwrite = TRUE
)

# Skip the README if you prefer
buildPacket(project_dir = "atacseq_analysis", include_readme = FALSE)
```

### Including Supplementary CSVs

By default, `buildPacket()` bundles only the output files
(Excel workbooks, plots, etc.). Set
`include_supplementary = TRUE` to also include the raw
supplementary CSV files from `.locusPackRat/supplementary/`,
or pass a character vector to include specific tables:

```
# Include all supplementary CSVs
buildPacket(
  project_dir = "qtl_analysis",
  include_supplementary = TRUE,
  overwrite = TRUE
)

# Include only specific supplementary tables
buildPacket(
  project_dir = "qtl_analysis",
  include_supplementary = c("mouseMine", "openTargets_diseases"),
  overwrite = TRUE
)
```

Supplementary files are placed in a `supplementary/`
subdirectory within the zip, and the auto-generated README lists them in
a separate “Included Supplementary Files” table.

# Working Across Species and Genome Builds

## Human Gene Analysis with Mouse Orthologs

The package automatically handles orthology mapping between human and
mouse using the built-in coordinate files.

```
# Define human cancer genes
cancer_genes <- c("BRCA1", "BRCA2", "TP53", "EGFR", "KRAS", "MYC",
                 "PTEN", "RB1", "VHL", "APC", "ATM", "CDKN2A",
                 "MLH1", "MSH2", "BRAF", "PIK3CA", "ERBB2", "ALK")

human_genes <- data.table(
  gene_symbol = cancer_genes,
  cancer_type = c(rep("breast", 2), "multiple", "lung", "colorectal", "multiple",
                  "brain", "retinoblastoma", "kidney", "colorectal", "breast", "melanoma",
                  rep("colorectal", 2), "melanoma", "multiple", "breast", "lung"),
  mutation_frequency = round(runif(length(cancer_genes), 5, 80), 1)
)

# Initialize with automatic orthology mapping
initPackRat(
  data = human_genes,
  mode = "gene",
  species = "human",
  genome = "hg38",
  project_dir = "cancer_genes",
  force = TRUE
)
#> Initializing locusPackRat project...
#> Note: All data added to this project must use the hg38 genome build. Mixing genome builds will cause incorrect coordinate matching. Use rtracklayer::liftOver() to convert coordinates if needed.
#> Processing gene list...
#>   Matched all 18 genes to coordinates
#> Generating orthology information...
#> Saved gene data to cancer_genes/.locusPackRat/input/genes.csv
#> Saved orthology data to cancer_genes/.locusPackRat/input/orthology.csv
#> Created config file: cancer_genes/.locusPackRat/config.json
#> 
#> locusPackRat project initialized successfully!
#> Mode: gene | Species: human | Genome: hg38
#> Processed 18 genes

# Add clinical significance data
clinical_data <- data.table(
  gene_symbol = cancer_genes,
  pathogenicity = sample(c("pathogenic", "likely_pathogenic", "VUS"),
                        length(cancer_genes), replace = TRUE,
                        prob = c(0.5, 0.3, 0.2)),
  FDA_approved_drug = sample(c(TRUE, FALSE), length(cancer_genes),
                            replace = TRUE, prob = c(0.3, 0.7)),
  clinical_trials = sample(0:10, length(cancer_genes), replace = TRUE)
)

addRatTable(
  data = clinical_data,
  table_name = "clinical_significance",
  link_type = "gene",
  link_by = "gene_symbol",
  project_dir = "cancer_genes"
)
#> Adding supplementary table to human hg38 project...
#> Linking data by gene_symbol...
#> Saved supplementary table to cancer_genes/.locusPackRat/supplementary/clinical_significance.csv
#> Linked 18 of 18 input rows
#> Updated config file

# Generate cross-species analysis workbook
makeGeneSheet(
  format = "excel",
  output_file = "cancer_genes_analysis.xlsx",
  split_by = "criteria",
  split_criteria = list(
    "High_Frequency" = "mutation_frequency > 30",
    "FDA_Targets" = "FDA_approved_drug == TRUE",
    "Breast_Cancer" = "cancer_type == 'breast'",
    "With_Mouse_Ortholog" = "!is.na(mouse_gene_symbol)"
  ),
  include_supplementary = TRUE,
  project_dir = "cancer_genes"
)
#> Generating gene sheet from cancer_genes/.locusPackRat...
#> Found 1 supplementary files: clinical_significance.csv
#> 
#>   Linking clinical_significance (Exact Match: gene_symbol)
#> Saved Excel to cancer_genes/.locusPackRat/output/cancer_genes_analysis.xlsx
```

## Converting Between Genome Coordinates

In some cases, users may have data that use older genome builds
(e.g. mm9) and wish to incorporate them into a project based around a
newer build (e.g. mm39).

Although LocusPackRat does not have a built-in function to accomodate
other builds, the liftOver package, paired with chain files drawn from
the UCSC genome browser, can easily do these conversions.

```
library(liftOver)
mm9_ncRNA = read.csv(system.file("extdata", "sample_mm9_ncRNA.csv", package = "locusPackRat"))
chain = import.chain( system.file("extdata", "mm9ToMm39.over.chain", package = "locusPackRat"))

#convert table to GRanges object.  Note that the chain files normally want
#chromosomes in 'chr1' format vs '1' format.  So you will need to convert.
mm9_ncRNA$chr=paste0("chr",mm9_ncRNA$chr)
mm9_ncRNA=makeGRangesFromDataFrame(mm9_ncRNA,keep.extra.columns = T)

mm39_ncRNA=unlist(liftOver(mm9_ncRNA,chain = chain))
mm39_ncRNA=as.data.frame(mm39_ncRNA)
mm39_ncRNA$seqnames=gsub("chr","",mm39_ncRNA$seqnames)

#Your data are now ready to be added to a locusPackRat project
```

# Working with Projects

## List Available Tables

```
# View supplementary tables in each project
listPackRatTables("rnaseq_analysis", full_info = TRUE)
#> Found 6 supplementary table(s):
#>   - mouse_phenotypes: 3592 rows with 7 cols, linked by 'gene_symbol'
#>   - ot_constraints: 9 rows with 10 cols, linked by 'gene_symbol'
#>   - ot_diseases: 389 rows with 6 cols, linked by 'gene_symbol'
#>   - ot_tractability: 168 rows with 6 cols, linked by 'gene_symbol'
#>   - pathway_annotations: 79 rows with 4 cols, linked by 'gene_symbol'
#>   - tissue_expression: 150 rows with 10 cols, linked by 'gene_symbol'
#> Printing full column names for 6 supplementary table(s)
#> Columns in tissue_expression:
#> gene_symbol ; ensembl_id ; brain ; liver ; heart ; kidney ; lung ; muscle ; max_tissue ; tissue_specific
#> Columns in pathway_annotations:
#> gene_symbol ; ensembl_id ; pathway ; evidence
#> Columns in mouse_phenotypes:
#> gene_symbol ; ensembl_id ; mgi_id ; mp_id ; phenotype ; pubmed_id ; description
#> Columns in ot_diseases:
#> gene_symbol ; ensembl_id ; human_ensembl_id ; disease_id ; disease_name ; score
#> Columns in ot_constraints:
#> gene_symbol ; ensembl_id ; human_ensembl_id ; constraintType ; exp ; obs ; score ; oe ; oeLower ; oeUpper
#> Columns in ot_tractability:
#> gene_symbol ; ensembl_id ; human_ensembl_id ; label ; modality ; value
#> Completed
#>             table_name table_abbr link_type     link_by n_rows n_cols
#>                 <char>     <char>    <char>      <char>  <int>  <int>
#> 1:    mouse_phenotypes         mm      gene gene_symbol   3592      7
#> 2:      ot_constraints        otc      gene gene_symbol      9     10
#> 3:         ot_diseases        otd      gene gene_symbol    389      6
#> 4:     ot_tractability        ott      gene gene_symbol    168      6
#> 5: pathway_annotations       <NA>      gene gene_symbol     79      4
#> 6:   tissue_expression         te      gene gene_symbol    150     10
#>    date_added
#>        <char>
#> 1: 2026-02-18
#> 2: 2026-02-18
#> 3: 2026-02-18
#> 4: 2026-02-18
#> 5: 2026-02-18
#> 6: 2026-02-18
listPackRatTables("atacseq_analysis", full_info = FALSE)
#> Found 7 supplementary table(s):
#>   - chromatin_states: 200 rows with 5 cols, linked by 'chr,start,end'
#>   - differential_accessibility: 200 rows with 9 cols, linked by 'chr,start,end'
#>   - mouse_phenotypes: 1404 rows with 6 cols, linked by 'gene_symbol'
#>   - ot_constraints: 45 rows with 9 cols, linked by 'gene_symbol'
#>   - ot_diseases: 3192 rows with 5 cols, linked by 'gene_symbol'
#>   - ot_tractability: 420 rows with 5 cols, linked by 'gene_symbol'
#>   - tf_motifs: 150 rows with 6 cols, linked by 'chr,start,end'
#>                    table_name table_abbr link_type       link_by n_rows n_cols
#>                        <char>     <char>    <char>        <char>  <int>  <int>
#> 1:           chromatin_states         cs    region chr,start,end    200      5
#> 2: differential_accessibility         da    region chr,start,end    200      9
#> 3:           mouse_phenotypes         mm      gene   gene_symbol   1404      6
#> 4:             ot_constraints        otc      gene   gene_symbol     45      9
#> 5:                ot_diseases        otd      gene   gene_symbol   3192      5
#> 6:            ot_tractability        ott      gene   gene_symbol    420      5
#> 7:                  tf_motifs  tf_motifs    region chr,start,end    150      6
#>    date_added
#>        <char>
#> 1: 2026-02-18
#> 2: 2026-02-18
#> 3: 2026-02-18
#> 4: 2026-02-18
#> 5: 2026-02-18
#> 6: 2026-02-18
#> 7: 2026-02-18
```

## Remove a Table

```
removeRatTable("chromatin_states", project_dir = "atacseq_analysis")
```

## Project Configuration

Each project contains a `config.json` file with project
metadata:

```
# Load project configuration
config <- jsonlite::read_json("rnaseq_analysis/.locusPackRat/config.json")
str(config)
```

## Output Options

### CSV Format

```
makeGeneSheet(
  format = "csv",
  output_file = "results.csv",
  include_supplementary = TRUE,
  project_dir = "rnaseq_analysis"
)
```

### Filtered Output

```
makeGeneSheet(
  format = "csv",
  output_file = "significant_genes.csv",
  filter_expr = "padj < 0.05 & abs(log2FC) > 1",
  include_supplementary = TRUE,
  project_dir = "rnaseq_analysis"
)
```

### Selective Table Inclusion

```
makeGeneSheet(
  format = "excel",
  output_file = "selected_data.xlsx",
  include_supplementary = c("tissue_expression", "pathway_annotations"),
  project_dir = "rnaseq_analysis"
)
```

# Converting Genome Builds

All data added to a locusPackRat project must use the same genome
build (e.g., mm39, hg38). If you have data in a different build, use
`rtracklayer::liftOver()` to convert coordinates before
adding them to the project.

```
# Example: Convert mm10 coordinates to mm39
library(rtracklayer)
library(GenomicRanges)

# Load a chain file for the conversion
# Chain files are available from UCSC:
# https://hgdownload.cse.ucsc.edu/goldenpath/mm10/liftOver/mm10ToMm39.over.chain.gz
chain <- import.chain("mm10ToMm39.over.chain")

# Create a GRanges object from your data
# Suppose you have eQTL data in mm10 coordinates
eqtl_mm10 <- data.table(
  chr = c("chr3", "chr3", "chr3"),
  start = c(131000000, 135000000, 142000000),
  end = c(131001000, 135001000, 142001000),
  gene = c("Cisd2", "Manba", "Pdlim5"),
  lod = c(5.2, 4.8, 3.1)
)

gr_mm10 <- GRanges(
  seqnames = eqtl_mm10$chr,
  ranges = IRanges(start = eqtl_mm10$start, end = eqtl_mm10$end),
  gene = eqtl_mm10$gene,
  lod = eqtl_mm10$lod
)

# Perform liftOver
gr_mm39 <- liftOver(gr_mm10, chain)

# Convert back to data.table — liftOver returns a GRangesList
# (one element per input range, since some ranges may map to multiple locations)
gr_mm39_unlisted <- unlist(gr_mm39)

eqtl_mm39 <- data.table(
  chr = as.character(seqnames(gr_mm39_unlisted)),
  start = start(gr_mm39_unlisted),
  end = end(gr_mm39_unlisted),
  gene = gr_mm39_unlisted$gene,
  lod = gr_mm39_unlisted$lod
)

# Remove "chr" prefix if your project uses UCSC-style names without it
eqtl_mm39[, chr := gsub("^chr", "", chr)]

# Now add to your mm39 locusPackRat project
addRatTable(
  data = eqtl_mm39,
  table_name = "eqtl_lifted",
  abbreviation = "eql",
  link_type = "region",
  link_by = "chr,start,end",
  project_dir = "qtl_analysis"
)
```

# Session Information

```
sessionInfo()
#> R version 4.5.2 (2025-10-31)
#> Platform: x86_64-conda-linux-gnu
#> Running under: Red Hat Enterprise Linux 9.7 (Plow)
#> 
#> Matrix products: default
#> BLAS/LAPACK: /nas/longleaf/home/bgural/mambaforge/envs/packrat_dev/lib/libopenblasp-r0.3.30.so;  LAPACK version 3.12.0
#> 
#> locale:
#>  [1] LC_CTYPE=en_US.UTF-8       LC_NUMERIC=C              
#>  [3] LC_TIME=en_US.UTF-8        LC_COLLATE=en_US.UTF-8    
#>  [5] LC_MONETARY=en_US.UTF-8    LC_MESSAGES=en_US.UTF-8   
#>  [7] LC_PAPER=en_US.UTF-8       LC_NAME=C                 
#>  [9] LC_ADDRESS=C               LC_TELEPHONE=C            
#> [11] LC_MEASUREMENT=en_US.UTF-8 LC_IDENTIFICATION=C       
#> 
#> time zone: America/New_York
#> tzcode source: system (glibc)
#> 
#> attached base packages:
#> [1] stats     graphics  grDevices utils     datasets  methods   base     
#> 
#> other attached packages:
#> [1] jsonlite_2.0.0     httr_1.4.8         data.table_1.17.8  locusPackRat_0.6.2
#> 
#> loaded via a namespace (and not attached):
#>  [1] tidyselect_1.2.1                         
#>  [2] blob_1.2.4                               
#>  [3] dplyr_1.2.0                              
#>  [4] farver_2.1.2                             
#>  [5] Biostrings_2.74.1                        
#>  [6] S7_0.2.1                                 
#>  [7] bitops_1.0-9                             
#>  [8] fastmap_1.2.0                            
#>  [9] RCurl_1.98-1.17                          
#> [10] GenomicAlignments_1.42.0                 
#> [11] XML_3.99-0.20                            
#> [12] digest_0.6.39                            
#> [13] lifecycle_1.0.5                          
#> [14] plyranges_1.26.0                         
#> [15] KEGGREST_1.46.0                          
#> [16] RSQLite_2.4.4                            
#> [17] magrittr_2.0.4                           
#> [18] compiler_4.5.2                           
#> [19] rlang_1.1.7                              
#> [20] sass_0.4.10                              
#> [21] tools_4.5.2                              
#> [22] yaml_2.3.12                              
#> [23] rtracklayer_1.66.0                       
#> [24] knitr_1.51                               
#> [25] S4Arrays_1.6.0                           
#> [26] bit_4.6.0                                
#> [27] curl_7.0.0                               
#> [28] DelayedArray_0.32.0                      
#> [29] RColorBrewer_1.1-3                       
#> [30] abind_1.4-8                              
#> [31] BiocParallel_1.40.2                      
#> [32] withr_3.0.2                              
#> [33] purrr_1.2.1                              
#> [34] BiocGenerics_0.56.0                      
#> [35] grid_4.5.2                               
#> [36] stats4_4.5.2                             
#> [37] Rhdf5lib_1.28.0                          
#> [38] ggplot2_4.0.2                            
#> [39] scales_1.4.0                             
#> [40] SummarizedExperiment_1.36.0              
#> [41] cli_3.6.5                                
#> [42] rmarkdown_2.30                           
#> [43] crayon_1.5.3                             
#> [44] generics_0.1.4                           
#> [45] otel_0.2.0                               
#> [46] rjson_0.2.23                             
#> [47] DBI_1.2.3                                
#> [48] cachem_1.1.0                             
#> [49] rhdf5_2.50.2                             
#> [50] zlibbioc_1.52.0                          
#> [51] parallel_4.5.2                           
#> [52] AnnotationDbi_1.68.0                     
#> [53] ggplotify_0.1.3                          
#> [54] XVector_0.46.0                           
#> [55] restfulr_0.0.16                          
#> [56] matrixStats_1.5.0                        
#> [57] yulab.utils_0.2.4                        
#> [58] vctrs_0.7.1                              
#> [59] Matrix_1.7-4                             
#> [60] gridGraphics_0.5-1                       
#> [61] IRanges_2.40.1                           
#> [62] S4Vectors_0.48.0                         
#> [63] bit64_4.6.0-1                            
#> [64] GenomicFeatures_1.58.0                   
#> [65] strawr_0.0.92                            
#> [66] jquerylib_0.1.4                          
#> [67] glue_1.8.0                               
#> [68] TxDb.Mmusculus.UCSC.mm39.knownGene_3.20.0
#> [69] org.Mm.eg.db_3.20.0                      
#> [70] plotgardener_1.12.0                      
#> [71] codetools_0.2-20                         
#> [72] stringi_1.8.7                            
#> [73] gtable_0.3.6                             
#> [74] GenomeInfoDb_1.42.3                      
#> [75] GenomicRanges_1.58.0                     
#> [76] BiocIO_1.16.0                            
#> [77] UCSC.utils_1.2.0                         
#> [78] tibble_3.3.1                             
#> [79] pillar_1.11.1                            
#> [80] rappdirs_0.3.4                           
#> [81] htmltools_0.5.9                          
#> [82] rhdf5filters_1.18.1                      
#> [83] GenomeInfoDbData_1.2.13                  
#> [84] R6_2.6.1                                 
#> [85] evaluate_1.0.5                           
#> [86] lattice_0.22-9                           
#> [87] Biobase_2.66.0                           
#> [88] png_0.1-8                                
#> [89] Rsamtools_2.22.0                         
#> [90] openxlsx_4.2.8.1                         
#> [91] memoise_2.0.1                            
#> [92] bslib_0.10.0                             
#> [93] Rcpp_1.1.1                               
#> [94] zip_2.3.3                                
#> [95] SparseArray_1.6.2                        
#> [96] xfun_0.56                                
#> [97] fs_1.6.6                                 
#> [98] MatrixGenerics_1.18.1                    
#> [99] pkgconfig_2.0.3
```
