# Supplementary material for: LocusPackRat: an R package to support prioritizing candidate genes from large GWAS intervals with standardized evidence aggregation
Source: G3 (Bethesda). 2026 Mar 28;16(6):jkag081. doi: 10.1093/g3journal/jkag081 (PMC13232493; doi:10.1093/g3journal/jkag081)
Supplement: jkag081_Supplementary_Data [file jkag081_supplementary_data.zip › Supplementary_File_2_G3-2026-406637.html]

Region-Level QTL Analysis with Open Targets Integration


# Region-Level QTL Analysis with Open Targets Integration

#### Brian Gural, Todd Kimball, Anh Luu, Christoph D. Rau

#### 2026-02-18

- Overview
- Setup
- Define QTL
  Regions
- Initialize
  Project
- Add Simulated LOD Data
- Query Open Targets
  Platform
- Query Open Targets QTL Data
  - QTL Studies
    Table
  - QTL Credible Sets Table
- Examine Project Structure
- Generate Locus Zoom Plots
- Export
  Results
- Notes and
  Caveats
  - API Rate
    Limits
  - Species
    Handling
  - Offline
    Usage
  - L2G Scores
- Session Info
- Cleanup

# Overview

This vignette demonstrates a region-level QTL analysis workflow using
`locusPackRat`. We focus on three human genomic regions (~2
MB each) that have documented eQTL and pQTL data in Open Targets: 1.
**ZNF586 locus** (chr19): Region with extensive eQTL data
2. **APIP locus** (chr11): Region with expression QTL
associations 3. **CLEC19A locus** (chr16): Region with
protein QTL data

We showcase the Open Targets Platform integration functions:

- `queryOpenTargets()`: Disease associations, genetic
  constraints, and tractability
- `queryOpenTargetsQTL()`: eQTL and pQTL data from
  fine-mapped credible sets

# Setup

```
library(locusPackRat)
library(data.table)
```

# Define QTL Regions

We define three regions of approximately 2 MB each, selected because
they have documented eQTL and pQTL data in the Open Targets
Platform.

```
qtl_regions <- data.table(
  chr = c("19", "11", "16"),
  start = c(56769655, 33875225, 18285731),
  end = c(58769655, 35875225, 20285731),
  region_id = c("ZNF586_locus", "APIP_locus", "CLEC19A_locus"),
  peak_lod = c(8.2, 6.5, 7.1),
  trait = c("gene_expression", "gene_expression", "protein_levels")
)

# Display region summary
qtl_regions[, .(
  region_id,
  chr,
  size_kb = round((end - start) / 1000),
  trait
)]
#>        region_id    chr size_kb           trait
#>           <char> <char>   <num>          <char>
#> 1:  ZNF586_locus     19    2000 gene_expression
#> 2:    APIP_locus     11    2000 gene_expression
#> 3: CLEC19A_locus     16    2000  protein_levels
```

# Initialize Project

Initialize a region-mode project with human hg38 coordinates.

```
initPackRat(
  data = qtl_regions,
  mode = "region",
  species = "human",
  genome = "hg38",
  project_dir = "qtl_opentargets_demo",
  force = TRUE
)
```

# Add Simulated LOD Data

For demonstration purposes, we generate simulated QTL scan data with
Gaussian peaks centered in each region. In a real analysis, this would
be replaced with actual scan results from qtl2 or similar software.

```
# Helper function to generate realistic LOD curves
generate_lod_data <- function(regions, points_per_region = 500,
                              noise_sd = 0.3) {
  lod_list <- lapply(seq_len(nrow(regions)), function(i) {
    region <- regions[i, ]
    positions <- seq(region$start, region$end, length.out = points_per_region)

    # Create Gaussian peak centered at midpoint
    peak_pos <- (region$start + region$end) / 2
    peak_width <- (region$end - region$start) / 6

    # Base LOD from Gaussian curve + noise
    base_lod <- region$peak_lod *
      exp(-((positions - peak_pos)^2) / (2 * peak_width^2))
    lod_values <- pmax(0, base_lod + rnorm(length(positions), 0, noise_sd))

    data.table(
      chr = region$chr,
      pos = as.integer(positions),
      lod = round(lod_values, 3),
      region_id = region$region_id,
      trait = region$trait
    )
  })

  rbindlist(lod_list)
}

# Generate LOD data
set.seed(42)
lod_scan <- generate_lod_data(qtl_regions)

# Preview
head(lod_scan)
#>       chr      pos   lod    region_id           trait
#>    <char>    <int> <num>       <char>          <char>
#> 1:     19 56769655 0.502 ZNF586_locus gene_expression
#> 2:     19 56773663 0.000 ZNF586_locus gene_expression
#> 3:     19 56777671 0.207 ZNF586_locus gene_expression
#> 4:     19 56781679 0.291 ZNF586_locus gene_expression
#> 5:     19 56785687 0.226 ZNF586_locus gene_expression
#> 6:     19 56789695 0.077 ZNF586_locus gene_expression
```

```
# Add scan data to project
addRatTable(
  data = lod_scan,
  table_name = "qtl_scan",
  link_type = "point",
  project_dir = "qtl_opentargets_demo"
)
```

# Query Open Targets Platform

The Open Targets Platform provides comprehensive gene annotations
including disease associations, genetic constraints (gnomAD), and drug
tractability assessments.

```
queryOpenTargets(
  project_dir = "qtl_opentargets_demo",
  data_types = c("diseases", "constraints", "tractability"),
  disease_limit = 100,  # Limit for vignette performance
  verbose = TRUE
)
```

# Query Open Targets QTL Data

The `queryOpenTargetsQTL()` function retrieves fine-mapped
eQTL and pQTL data from credible sets, including locus-to-gene (L2G)
prediction scores. The function queries by genomic region, making it
suitable for both gene-mode and region-mode projects.

```
qtl_results <- queryOpenTargetsQTL(
  project_dir = "qtl_opentargets_demo",
  study_types = c("eqtl", "pqtl"),
  include_l2g = TRUE,
  min_l2g_score = 0.5,
  verbose = TRUE
)
#> Project Config: human - hg38 (mode: region)
#> Querying Open Targets QTL for 3 regions...
#> Study types: EQTL, PQTL
#>   Processing batch 1/1 (3 regions)...
#> Adding supplementary table to human hg38 project...
#> Linking data by gene_symbol...
#> Saved supplementary table to qtl_opentargets_demo/.locusPackRat/supplementary/ot_qtl_studies.csv
#> Linked 169 of 169 input rows
#> Updated config file
#>   Saved ot_qtl_studies with 169 rows
#> Adding supplementary table to human hg38 project...
#> Linking data by gene_symbol...
#> Saved supplementary table to qtl_opentargets_demo/.locusPackRat/supplementary/ot_qtl_credible_sets.csv
#> Linked 191 of 191 input rows
#> Updated config file
#>   Saved ot_qtl_credible_sets with 191 rows
```

## QTL Studies Table

The studies table contains metadata about each QTL study, including
tissue/cell type (biosample), sample size, and the target gene.

```
if (!is.null(qtl_results$studies) && nrow(qtl_results$studies) > 0) {
  studies_dt <- qtl_results$studies
  cat("QTL Studies table:", nrow(studies_dt), "rows\n\n")

  # Select key columns for display
  display_cols <- intersect(
    c("study_id", "study_type", "biosample_name",
      "n_samples", "target_gene_symbol"),
    names(studies_dt)
  )

  cat("Head of studies table:\n")
  print(head(studies_dt[, ..display_cols], 5))

  cat("\nTail of studies table:\n")
  print(tail(studies_dt[, ..display_cols], 5))
} else {
  cat("No QTL studies found for the specified regions.\n")
}
#> QTL Studies table: 169 rows
#> 
#> Head of studies table:
#>                                          study_id study_type
#>                                            <char>     <char>
#> 1:          blueprint_ge_monocyte_ensg00000083828       eqtl
#> 2: gtex_tx_heart_atrial_appendage_enst00000598183       eqtl
#> 3:          gtex_ge_artery_tibial_ensg00000083828       eqtl
#> 4:       gtex_tx_skin_sun_exposed_enst00000598183       eqtl
#> 5:          gtex_tx_adrenal_gland_enst00000651718       eqtl
#>                                     biosample_name n_samples target_gene_symbol
#>                                             <char>    <lgcl>             <char>
#> 1: CD14-positive, CD16-negative classical monocyte        NA             ZNF586
#> 2:                   right atrium auricular region        NA             ZNF586
#> 3:                                   tibial artery        NA             ZNF586
#> 4:                                    skin of body        NA             ZNF586
#> 5:                                   adrenal gland        NA             ZNF586
#> 
#> Tail of studies table:
#>                                                 study_id study_type
#>                                                   <char>     <char>
#> 1:            quach_2016_ge_monocyte_lps_ensg00000261210       eqtl
#> 2: nedelec_2016_ge_macrophage_salmonella_ensg00000261210       eqtl
#> 3:      nedelec_2016_ge_macrophage_naive_ensg00000261210       eqtl
#> 4:            quach_2016_ge_monocyte_iav_ensg00000261210       eqtl
#> 5:           quach_2016_ge_monocyte_r848_ensg00000261210       eqtl
#>                                     biosample_name n_samples target_gene_symbol
#>                                             <char>    <lgcl>             <char>
#> 1: CD14-positive, CD16-negative classical monocyte        NA            CLEC19A
#> 2:                                      macrophage        NA            CLEC19A
#> 3:                                      macrophage        NA            CLEC19A
#> 4: CD14-positive, CD16-negative classical monocyte        NA            CLEC19A
#> 5: CD14-positive, CD16-negative classical monocyte        NA            CLEC19A
```

## QTL Credible Sets Table

The credible sets table contains fine-mapped QTL associations with
lead variants, effect sizes, and L2G scores linking variants to their
most likely causal genes.

```
credsets_dt <- qtl_results$credible_sets
if (!is.null(credsets_dt) && nrow(credsets_dt) > 0) {
  cat("QTL Credible Sets table:", nrow(credsets_dt), "rows\n\n")

  # Select key columns for display (L2G columns omitted -- see note below)
  display_cols <- intersect(
    c("study_id", "study_type", "chromosome", "position", "rsid",
      "beta", "pvalue_mantissa", "pvalue_exponent"),
    names(credsets_dt)
  )

  cat("Head of credible sets table:\n")
  print(head(credsets_dt[, ..display_cols], 5))

  cat("\nTail of credible sets table:\n")
  print(tail(credsets_dt[, ..display_cols], 5))

  # Summary statistics
  cat("\n\nSummary by study type:\n")
  print(credsets_dt[, .N, by = study_type])

  cat("\nNote: L2G (locus-to-gene) prediction scores are primarily available",
      "for GWAS credible sets.\n",
      "molQTL studies (eQTL/pQTL) typically return empty L2G predictions",
      "because the\n",
      "QTL target gene is already known from the study design.",
      "The l2g_gene_symbol and\n",
      "l2g_score columns are retained in the stored data table",
      "for use with GWAS queries.\n")
} else {
  cat("No QTL credible sets found for the specified regions.\n")
}
#> QTL Credible Sets table: 191 rows
#> 
#> Head of credible sets table:
#>                                          study_id study_type chromosome
#>                                            <char>     <char>     <char>
#> 1:          blueprint_ge_monocyte_ensg00000083828       eqtl         19
#> 2: gtex_tx_heart_atrial_appendage_enst00000598183       eqtl         19
#> 3:          gtex_ge_artery_tibial_ensg00000083828       eqtl         19
#> 4:       gtex_tx_skin_sun_exposed_enst00000598183       eqtl         19
#> 5:          gtex_tx_adrenal_gland_enst00000651718       eqtl         19
#>    position       rsid      beta pvalue_mantissa pvalue_exponent
#>       <int>     <list>     <num>           <num>           <int>
#> 1: 57819143 rs35962362  0.743070           1.148              -7
#> 2: 57821405  rs7255684  0.517417           7.156             -11
#> 3: 57829516  rs2115384  0.481659           7.023             -14
#> 4: 57849960 rs28374851  0.406202           4.047             -12
#> 5: 57693820  rs9304799 -0.301088           1.022              -6
#> 
#> Tail of credible sets table:
#>                                                 study_id study_type chromosome
#>                                                   <char>     <char>     <char>
#> 1:      nedelec_2016_ge_macrophage_naive_ensg00000261210       eqtl         16
#> 2:            quach_2016_ge_monocyte_iav_ensg00000261210       eqtl         16
#> 3:      nedelec_2016_ge_macrophage_naive_ensg00000261210       eqtl         16
#> 4:           quach_2016_ge_monocyte_r848_ensg00000261210       eqtl         16
#> 5: nedelec_2016_ge_macrophage_salmonella_ensg00000261210       eqtl         16
#>    position        rsid      beta pvalue_mantissa pvalue_exponent
#>       <int>      <list>     <num>           <num>           <int>
#> 1: 19298899  rs66465307  0.787226           1.443              -7
#> 2: 19304838  rs58536649 -0.665561           5.067             -12
#> 3: 19304838  rs58536649 -0.808534           1.471             -14
#> 4: 19306095 rs111532635 -0.717730           3.351             -11
#> 5: 19306095 rs111532635 -0.663725           7.854             -14
#> 
#> 
#> Summary by study type:
#>    study_type     N
#>        <char> <int>
#> 1:       eqtl   191
#> 
#> Note: L2G (locus-to-gene) prediction scores are primarily available for GWAS credible sets.
#>  molQTL studies (eQTL/pQTL) typically return empty L2G predictions because the
#>  QTL target gene is already known from the study design. The l2g_gene_symbol and
#>  l2g_score columns are retained in the stored data table for use with GWAS queries.
```

# Examine Project Structure

Review all tables added to the project.

```
listPackRatTables(project_dir = "qtl_opentargets_demo", full_info = TRUE)
#> Found 6 supplementary table(s):
#>   - ot_constraints: 291 rows with 9 cols, linked by 'gene_symbol'
#>   - ot_diseases: 4371 rows with 5 cols, linked by 'gene_symbol'
#>   - ot_qtl_credible_sets: 191 rows with 28 cols, linked by 'gene_symbol'
#>   - ot_qtl_studies: 169 rows with 11 cols, linked by 'gene_symbol'
#>   - ot_tractability: 2744 rows with 5 cols, linked by 'gene_symbol'
#>   - qtl_scan: 1500 rows with 7 cols, linked by 'auto'
#> Printing full column names for 6 supplementary table(s)
#> Columns in qtl_scan:
#> chr ; pos ; lod ; region_id ; trait ; start ; end
#> Columns in ot_diseases:
#> gene_symbol ; human_ensembl_id ; disease_id ; disease_name ; score
#> Columns in ot_constraints:
#> gene_symbol ; human_ensembl_id ; constraintType ; exp ; obs ; score ; oe ; oeLower ; oeUpper
#> Columns in ot_tractability:
#> gene_symbol ; human_ensembl_id ; label ; modality ; value
#> Columns in ot_qtl_studies:
#> gene_symbol ; target_gene_id ; study_id ; study_type ; trait ; project_id ; biosample_id ; biosample_name ; n_samples ; has_sumstats ; target_gene_symbol
#> Columns in ot_qtl_credible_sets:
#> gene_symbol ; qtl_gene_id ; study_locus_id ; study_id ; study_type ; is_trans_qtl ; chromosome ; position ; region ; locus_start ; locus_end ; lead_variant_id ; ref_allele ; alt_allele ; rsid ; pvalue_mantissa ; pvalue_exponent ; beta ; se ; eaf ; finemapping_method ; confidence ; credible_set_index ; biosample_id ; biosample_name ; l2g_gene_id ; l2g_gene_symbol ; l2g_score
#> Completed
#>              table_name table_abbr link_type     link_by n_rows n_cols
#>                  <char>     <char>    <char>      <char>  <int>  <int>
#> 1:       ot_constraints        otc      gene gene_symbol    291      9
#> 2:          ot_diseases        otd      gene gene_symbol   4371      5
#> 3: ot_qtl_credible_sets       otqc      gene gene_symbol    191     28
#> 4:       ot_qtl_studies       otqs      gene gene_symbol    169     11
#> 5:      ot_tractability        ott      gene gene_symbol   2744      5
#> 6:             qtl_scan       <NA>     point        auto   1500      7
#>    date_added
#>        <char>
#> 1: 2026-02-18
#> 2: 2026-02-18
#> 3: 2026-02-18
#> 4: 2026-02-18
#> 5: 2026-02-18
#> 6: 2026-02-18
```

# Generate Locus Zoom Plots

Create publication-quality locus zoom plots for each region. These
plots show the QTL scan results alongside gene annotations.

**Note:** The gene annotation plots require Bioconductor
annotation packages. Install them with:

```
BiocManager::install(c("TxDb.Hsapiens.UCSC.hg38.knownGene", "org.Hs.eg.db"))
```

```
# ZNF586 locus - expression QTL region
generateLocusZoomPlot(
  region_id = "ZNF586_locus",
  project_dir = "qtl_opentargets_demo",
  scan_table = "qtl_scan",
  width = 8,
  height = 5,
  highlight_genes = c("ZNF586"),
  threshold = 5,
  output_file = "ZNF586_locus_zoom.png"
)
knitr::include_graphics(
  file.path("qtl_opentargets_demo", ".locusPackRat", "output", "ZNF586_locus_zoom.png")
)
```

```
# APIP locus - expression QTL region
generateLocusZoomPlot(
  region_id = "APIP_locus",
  project_dir = "qtl_opentargets_demo",
  scan_table = "qtl_scan",
  width = 8,
  height = 5,
  highlight_genes = c("APIP"),
  threshold = 5,
  output_file = "APIP_locus_zoom.png"
)
knitr::include_graphics(
  file.path("qtl_opentargets_demo", ".locusPackRat", "output", "APIP_locus_zoom.png")
)
```

```
# CLEC19A locus - protein QTL region
generateLocusZoomPlot(
  region_id = "CLEC19A_locus",
  project_dir = "qtl_opentargets_demo",
  scan_table = "qtl_scan",
  width = 8,
  height = 5,
  highlight_genes = c("CLEC19A"),
  threshold = 5,
  output_file = "CLEC19A_locus_zoom.png"
)
knitr::include_graphics(
  file.path("qtl_opentargets_demo", ".locusPackRat", "output", "CLEC19A_locus_zoom.png")
)
```

# Export Results

Generate a multi-sheet Excel workbook with filtered views of the
integrated data.

```
makeGeneSheet(
  format = "excel",
  output_file = "qtl_opentargets_results.xlsx",
  split_by = "criteria",
  prefix_mode = "collision",
  split_criteria = list(
    "All_Genes" = "TRUE",
    "High_LOD" = "peak_lod > 7",
    "Druggable" = "!is.na(ott_label)",
    "Constrained" = "otc_constraintType == 'lof' & otc_oe < 0.35"
  ),
  project_dir = "qtl_opentargets_demo"
)
```

# Notes and Caveats

## API Rate Limits

The Open Targets API has rate limits. For large gene sets,
consider:

- Using the `limit` parameter for testing
- Running queries during off-peak hours
- Caching results locally

## Species Handling

`queryOpenTargets()` and
`queryOpenTargetsQTL()` query human data. For mouse projects,
genes are automatically mapped to human orthologs before querying.

## Offline Usage

When running without internet access (or on CRAN), the API query
chunks are skipped. Pre-computed results can be loaded from local
files.

## L2G Scores

Locus-to-Gene (L2G) scores range from 0 to 1, where higher scores
indicate stronger evidence linking a variant to a gene. The default
threshold of 0.5 provides good specificity.

# Session Info

```
sessionInfo()
#> R version 4.5.2 (2025-10-31)
#> Platform: x86_64-conda-linux-gnu
#> Running under: Red Hat Enterprise Linux 9.7 (Plow)
#> 
#> Matrix products: default
#> BLAS/LAPACK: /nas/longleaf/home/bgural/mambaforge/envs/packrat_dev/lib/libopenblasp-r0.3.30.so;  LAPACK version 3.12.0
#> 
#> locale:
#>  [1] LC_CTYPE=en_US.UTF-8       LC_NUMERIC=C              
#>  [3] LC_TIME=en_US.UTF-8        LC_COLLATE=en_US.UTF-8    
#>  [5] LC_MONETARY=en_US.UTF-8    LC_MESSAGES=en_US.UTF-8   
#>  [7] LC_PAPER=en_US.UTF-8       LC_NAME=C                 
#>  [9] LC_ADDRESS=C               LC_TELEPHONE=C            
#> [11] LC_MEASUREMENT=en_US.UTF-8 LC_IDENTIFICATION=C       
#> 
#> time zone: America/New_York
#> tzcode source: system (glibc)
#> 
#> attached base packages:
#> [1] stats     graphics  grDevices utils     datasets  methods   base     
#> 
#> other attached packages:
#> [1] jsonlite_2.0.0     httr_1.4.8         data.table_1.17.8  locusPackRat_0.6.2
#> 
#> loaded via a namespace (and not attached):
#>   [1] tidyselect_1.2.1                         
#>   [2] blob_1.2.4                               
#>   [3] dplyr_1.2.0                              
#>   [4] farver_2.1.2                             
#>   [5] Biostrings_2.74.1                        
#>   [6] S7_0.2.1                                 
#>   [7] bitops_1.0-9                             
#>   [8] fastmap_1.2.0                            
#>   [9] RCurl_1.98-1.17                          
#>  [10] GenomicAlignments_1.42.0                 
#>  [11] XML_3.99-0.20                            
#>  [12] digest_0.6.39                            
#>  [13] lifecycle_1.0.5                          
#>  [14] plyranges_1.26.0                         
#>  [15] KEGGREST_1.46.0                          
#>  [16] TxDb.Hsapiens.UCSC.hg38.knownGene_3.20.0 
#>  [17] RSQLite_2.4.4                            
#>  [18] magrittr_2.0.4                           
#>  [19] compiler_4.5.2                           
#>  [20] rlang_1.1.7                              
#>  [21] sass_0.4.10                              
#>  [22] tools_4.5.2                              
#>  [23] yaml_2.3.12                              
#>  [24] rtracklayer_1.66.0                       
#>  [25] knitr_1.51                               
#>  [26] S4Arrays_1.6.0                           
#>  [27] bit_4.6.0                                
#>  [28] curl_7.0.0                               
#>  [29] DelayedArray_0.32.0                      
#>  [30] RColorBrewer_1.1-3                       
#>  [31] abind_1.4-8                              
#>  [32] BiocParallel_1.40.2                      
#>  [33] withr_3.0.2                              
#>  [34] purrr_1.2.1                              
#>  [35] BiocGenerics_0.56.0                      
#>  [36] grid_4.5.2                               
#>  [37] stats4_4.5.2                             
#>  [38] Rhdf5lib_1.28.0                          
#>  [39] ggplot2_4.0.2                            
#>  [40] scales_1.4.0                             
#>  [41] SummarizedExperiment_1.36.0              
#>  [42] cli_3.6.5                                
#>  [43] rmarkdown_2.30                           
#>  [44] crayon_1.5.3                             
#>  [45] generics_0.1.4                           
#>  [46] otel_0.2.0                               
#>  [47] rjson_0.2.23                             
#>  [48] DBI_1.2.3                                
#>  [49] cachem_1.1.0                             
#>  [50] rhdf5_2.50.2                             
#>  [51] zlibbioc_1.52.0                          
#>  [52] parallel_4.5.2                           
#>  [53] AnnotationDbi_1.68.0                     
#>  [54] ggplotify_0.1.3                          
#>  [55] XVector_0.46.0                           
#>  [56] restfulr_0.0.16                          
#>  [57] matrixStats_1.5.0                        
#>  [58] yulab.utils_0.2.4                        
#>  [59] vctrs_0.7.1                              
#>  [60] Matrix_1.7-4                             
#>  [61] gridGraphics_0.5-1                       
#>  [62] IRanges_2.40.1                           
#>  [63] S4Vectors_0.48.0                         
#>  [64] bit64_4.6.0-1                            
#>  [65] GenomicFeatures_1.58.0                   
#>  [66] strawr_0.0.92                            
#>  [67] jquerylib_0.1.4                          
#>  [68] glue_1.8.0                               
#>  [69] TxDb.Mmusculus.UCSC.mm39.knownGene_3.20.0
#>  [70] org.Mm.eg.db_3.20.0                      
#>  [71] plotgardener_1.12.0                      
#>  [72] codetools_0.2-20                         
#>  [73] stringi_1.8.7                            
#>  [74] gtable_0.3.6                             
#>  [75] GenomeInfoDb_1.42.3                      
#>  [76] GenomicRanges_1.58.0                     
#>  [77] BiocIO_1.16.0                            
#>  [78] UCSC.utils_1.2.0                         
#>  [79] tibble_3.3.1                             
#>  [80] pillar_1.11.1                            
#>  [81] rappdirs_0.3.4                           
#>  [82] htmltools_0.5.9                          
#>  [83] rhdf5filters_1.18.1                      
#>  [84] GenomeInfoDbData_1.2.13                  
#>  [85] R6_2.6.1                                 
#>  [86] evaluate_1.0.5                           
#>  [87] lattice_0.22-9                           
#>  [88] Biobase_2.66.0                           
#>  [89] png_0.1-8                                
#>  [90] Rsamtools_2.22.0                         
#>  [91] openxlsx_4.2.8.1                         
#>  [92] memoise_2.0.1                            
#>  [93] bslib_0.10.0                             
#>  [94] Rcpp_1.1.1                               
#>  [95] zip_2.3.3                                
#>  [96] SparseArray_1.6.2                        
#>  [97] org.Hs.eg.db_3.20.0                      
#>  [98] xfun_0.56                                
#>  [99] fs_1.6.6                                 
#> [100] MatrixGenerics_1.18.1                    
#> [101] pkgconfig_2.0.3
```

# Cleanup
