# Supplementary material for: LocusPackRat: an R package to support prioritizing candidate genes from large GWAS intervals with standardized evidence aggregation
Source: G3 (Bethesda). 2026 Mar 28;16(6):jkag081. doi: 10.1093/g3journal/jkag081 (PMC13232493; doi:10.1093/g3journal/jkag081)
Supplement: jkag081_Supplementary_Data [file jkag081_supplementary_data.zip › Supplementary_File_3_G3-2026-406637.html]

Querying GeneNetwork2 for Model Organism QTL Data


# Querying GeneNetwork2 for Model Organism QTL Data

#### Brian Gural, Todd Kimball, Anh Luu, Christoph D. Rau

#### 2026-02-18

- Overview
- Prerequisites
- GeneNetwork2 API Basics
- Example 1: BxD eQTL Data
  - List Available BxD
    Expression Datasets
  - Query Traits for a Specific
    Gene
  - Retrieve Trait Detail
  - Run QTL Mapping via GEMMA
- Example 2: BXD Published
  Phenotypes
- Integrating
  GeneNetwork2 Results into locusPackRat
  - Create a locusPackRat
    Project
  - Gene-Level Integration
  - Point-Level Integration
  - Project State After
    Integration
- Alternative: GNapi R
  Package
- Tips and Considerations
- Session Info

# Overview

This vignette demonstrates how to query the GeneNetwork2 REST API to retrieve
eQTL and phenotype data from model organism genetic reference
populations (GRPs), and how to integrate those results into a
`locusPackRat` project as additional evidence layers.

GeneNetwork2 hosts QTL mapping results, expression data, and
phenotype records for several GRPs, including:

- **BxD recombinant inbred lines** (mouse): extensive
  eQTL, pQTL, and phenotype data
- **Diversity Outbred (DO)** (mouse): high-resolution QTL
  mapping panels
- **Hybrid Mouse Diversity Panel (HMDP)**: cardiovascular
  and metabolic trait data
- **Other organisms**: rat (HxB/BxH), Drosophila, and
  Arabidopsis panels

Unlike Open Targets, which focuses on human GWAS data, GeneNetwork2
provides population-specific molecular QTL data that is directly
relevant to model organism studies. This makes it a valuable complement
to the human-centric annotations retrieved by
`queryOpenTargets()` and
`queryOpenTargetsQTL()`.

Live API calls are attempted first; if the GeneNetwork2 API is
unavailable, cached responses bundled with the package are used as a
fallback.

# Prerequisites

```
library(locusPackRat)
#> Warning: multiple methods tables found for 'union'
#> Warning: multiple methods tables found for 'intersect'
#> Warning: multiple methods tables found for 'setdiff'
#> Warning: multiple methods tables found for 'intersect'
#> Warning: multiple methods tables found for 'union'
#> Warning: multiple methods tables found for 'intersect'
#> Warning: multiple methods tables found for 'setdiff'
#> Warning: multiple methods tables found for 'union'
#> Warning: multiple methods tables found for 'intersect'
#> Warning: multiple methods tables found for 'setdiff'
library(data.table)
library(httr)
library(jsonlite)
```

# GeneNetwork2 API Basics

The GeneNetwork2 REST API is available at
`https://genenetwork.org/api/v_pre1/`. Key endpoints
include:

- **`/species`**: List available species
- **`/groups/{species}`**: List available
  populations (e.g., BXD, DO)
- **`/datasets/{group}`**: List available
  datasets for a population
- **`/traits/{dataset}`**: List traits within
  a dataset
- **`/trait/{dataset}/{probe_name}`**: Get
  detailed trait information

```
# Base URL for GeneNetwork2 API
gn2_base <- "https://genenetwork.org/api/v_pre1"

# Helper: fetch from API with cached fallback
gn_fetch_or_cache <- function(url, cache_file, timeout_sec = 30) {
  result <- tryCatch({
    resp <- httr::GET(url, httr::timeout(timeout_sec))
    httr::stop_for_status(resp)
    jsonlite::fromJSON(httr::content(resp, as = "text", encoding = "UTF-8"))
  }, error = function(e) NULL)

  if (!is.null(result) && length(result) > 0) {
    message("GeneNetwork API: live data retrieved")
    return(result)
  }

  cache_path <- system.file("extdata", "gn_cache", cache_file,
                            package = "locusPackRat")
  if (nzchar(cache_path) && file.exists(cache_path)) {
    message("GeneNetwork API: using cached data (API unavailable)")
    return(jsonlite::fromJSON(cache_path))
  }
  stop("GeneNetwork API unavailable and no cached data found for: ", cache_file)
}
```

# Example 1: BxD eQTL Data

## List Available BxD Expression Datasets

The BxD panel has dozens of expression datasets spanning multiple
tissues (brain, liver, heart, kidney, etc.) and platforms (microarray,
RNA-seq).

```
# Fetch all datasets for the BXD group
bxd_datasets <- gn_fetch_or_cache(
  paste0(gn2_base, "/datasets/BXD"),
  "bxd_datasets.json"
)
#> GeneNetwork API: live data retrieved

# The FullName column contains tissue and platform info
# Filter for expression datasets related to heart/cardiac tissue
heart_datasets <- bxd_datasets[grepl("Heart|Cardiac|heart|cardiac",
                                     bxd_datasets$FullName), ]

cat("Total BXD datasets:", nrow(bxd_datasets), "\n")
#> Total BXD datasets: 408
cat("Heart-related datasets:", nrow(heart_datasets), "\n\n")
#> Heart-related datasets: 9

# Preview heart datasets
display_cols <- intersect(names(heart_datasets),
                          c("Id", "FullName", "Short_Abbreviation"))
head(heart_datasets[, display_cols, drop = FALSE])
#>                                                                  FullName   Id
#> 276  EPFL/LISP BXD CD Heart Affy Mouse Gene 2.0 ST Gene Level (Jan14) RMA  485
#> 277 EPFL/LISP BXD HFD Heart Affy Mouse Gene 2.0 ST Gene Level (Jan14) RMA  486
#> 278  EPFL/LISP BXD CD Heart Affy Mouse Gene 2.0 ST Exon Level (Jan14) RMA  487
#> 279 EPFL/LISP BXD HFD Heart Affy Mouse Gene 2.0 ST Exon Level (Jan14) RMA  488
#> 385            NHLBI BXD Young Adult Heart CD RNA-Seq (Nov20) TMP Log2 ** 1023
#> 386        NHLBI BXD Young Adult Heart CD CMS RNA-Seq (Nov20) TMP Log2 ** 1024
#>                         Short_Abbreviation
#> 276                   EPFL-LISPBXDHeCD0114
#> 277                  EPFL-LISPBXDHeHFD0114
#> 278                 EPFL-LISPBXDHeCDEx0114
#> 279                EPFL-LISPBXDHeHFDEx0114
#> 385 NHLBI_BXD_Young_Adult_Heart_CD_RNA-Seq
#> 386 NHLBI_BXD_Young_Adult_Heart_CD_CMS_RNA
```

## Query Traits for a Specific Gene

To check whether a candidate gene has eQTL evidence in the BxD panel,
query traits by gene symbol within a specific dataset. The traits
endpoint returns all probes/traits; we filter to our gene of
interest.

```
# Query Cisd2 expression traits in a BXD heart dataset
dataset_name <- "HC_M2_0606_P"
gene_of_interest <- "Cisd2"

# Fetch traits (pre-filtered to Cisd2 in cached version)
cisd2_traits <- gn_fetch_or_cache(
  paste0(gn2_base, "/traits/", dataset_name),
  "cisd2_traits.json"
)
#> GeneNetwork API: live data retrieved

# The live endpoint returns ALL traits; filter to our gene
# The cached version is already pre-filtered
if (nrow(cisd2_traits) > 20) {
  # Live data -- filter by Symbol column
  cisd2_traits <- cisd2_traits[grepl(gene_of_interest, cisd2_traits$Symbol,
                                     ignore.case = TRUE), ]
}

cat("Found", nrow(cisd2_traits), "probes/traits for", gene_of_interest, "\n\n")
#> Found 4 probes/traits for Cisd2
print(cisd2_traits[, c("Name", "Symbol", "Description", "Chr", "Mb")])
#>               Name Symbol                Description Chr       Mb
#> 12747   1428441_at  Cisd2 CDGSH iron sulfur domain 2   3 135.4066
#> 13589   1429283_at  Cisd2 CDGSH iron sulfur domain 2   3 135.4104
#> 15298 1430992_s_at  Cisd2 CDGSH iron sulfur domain 2   3 135.4070
#> 15577   1431271_at  Cisd2 CDGSH iron sulfur domain 2   3 135.4254
```

Each row represents a microarray probe targeting Cisd2. The
`LRS` column gives the likelihood ratio statistic for the
best eQTL, and `Peak Chr` / `Peak Mb` indicate the
genomic position of the peak association.

## Retrieve Trait Detail

Get detailed information for a specific probe, including the additive
effect and peak association statistics.

```
# Get detail for the first (strongest) Cisd2 probe
probe_name <- cisd2_traits$Name[1]

trait_detail <- gn_fetch_or_cache(
  paste0(gn2_base, "/trait/", dataset_name, "/", probe_name),
  "cisd2_trait_detail.json"
)
#> GeneNetwork API: live data retrieved

cat("Probe:", trait_detail$name, "\n")
#> Probe: 1428441_at
cat("Gene:", trait_detail$symbol, "\n")
#> Gene: Cisd2
cat("Description:", trait_detail$description, "\n")
#> Description: CDGSH iron sulfur domain 2
cat("Chromosome:", trait_detail$chr, "at", trait_detail$mb, "Mb\n")
#> Chromosome: 3 at 135.4066 Mb
cat("Best LRS:", round(trait_detail$lrs, 2),
    "(LOD ~", round(trait_detail$lrs / 4.61, 2), ")\n")
#> Best LRS: 21.02 (LOD ~ 4.56 )
cat("Peak locus:", trait_detail$locus, "\n")
#> Peak locus: rs30689880
cat("Additive effect:", round(trait_detail$additive, 4), "\n")
#> Additive effect: -0.091
```

## Run QTL Mapping via GEMMA

GeneNetwork2 can perform on-the-fly QTL mapping using GEMMA
(Genome-wide Efficient Mixed Model Association). This returns LOD scores
across the genome.

```
# Request GEMMA mapping for the Cisd2 probe
# Note: this endpoint may take 30-60 seconds to return results
gemma_url <- paste0(gn2_base, "/mapping?trait_id=", probe_name,
                    "&db=", dataset_name,
                    "&method=gemma")

gemma_raw <- gn_fetch_or_cache(gemma_url, "cisd2_gemma.json", timeout_sec = 120)
#> GeneNetwork API: live data retrieved

# The API returns a nested list; extract the data frame
if (is.data.frame(gemma_raw)) {
  gemma_results <- gemma_raw
} else if (is.list(gemma_raw) && length(gemma_raw) > 0) {
  gemma_results <- gemma_raw[[1]]
} else {
  gemma_results <- data.frame()
}

if (nrow(gemma_results) > 0) {
  eqtl_scan <- data.table(
    chr = gemma_results$chr,
    pos = gemma_results$Mb * 1e6,  # Convert Mb to bp
    pos_mb = gemma_results$Mb,
    lod = gemma_results$lod_score,
    p_value = gemma_results$p_value,
    marker = gemma_results$name,
    gene_symbol = gene_of_interest,
    source = "GeneNetwork2_BxD"
  )

  # Summary of the scan
  cat("Total markers scanned:", nrow(eqtl_scan), "\n")
  cat("Significant positions (LOD > 3):", sum(eqtl_scan$lod > 3), "\n\n")

  # Show the top peaks
  top_peaks <- eqtl_scan[order(-lod)][1:5]
  print(top_peaks[, .(chr, pos_mb, lod, p_value, marker)])
}
#> Total markers scanned: 21056 
#> Significant positions (LOD > 3): 252 
#> 
#>       chr   pos_mb      lod      p_value         marker
#>    <char>    <num>    <num>        <num>         <char>
#> 1:      3 132.2330 6.795907 1.599900e-07     rs30689880
#> 2:      3 129.4835 6.718934 1.910142e-07 rsm10000001734
#> 3:      3 129.5100 6.718934 1.910142e-07     rs30507350
#> 4:      3 129.6428 6.718934 1.910142e-07     rs30261132
#> 5:      3 129.6450 6.718934 1.910142e-07   UNC030323436
```

The strongest eQTL for Cisd2 maps to chromosome 3, near the gene’s
own position – consistent with a *cis*-eQTL, which supports its
candidacy in a QTL study targeting this region.

# Example 2: BXD Published Phenotypes

GeneNetwork2 also hosts curated phenotype data for BXD strains.
Published phenotypes can be accessed through the `BXDPublish`
dataset, providing higher-level trait-QTL associations.

```
# Fetch BXD published phenotype traits
bxd_pheno <- tryCatch({
  gn_fetch_or_cache(
    paste0(gn2_base, "/traits/BXDPublish"),
    cache_file = NULL  # no cache for this endpoint
  )
}, error = function(e) {
  message("BXD published phenotypes unavailable: ", e$message)
  NULL
})
#> GeneNetwork API: live data retrieved

if (!is.null(bxd_pheno) && nrow(bxd_pheno) > 0) {
  cat("Total BXD published phenotype traits:", nrow(bxd_pheno), "\n\n")

  # Show a few example traits
  display_cols <- intersect(names(bxd_pheno), c("Id", "Name", "Description", "Symbol"))
  print(head(bxd_pheno[, display_cols, drop = FALSE], 10))
} else {
  cat("BXD published phenotype data not available via this API endpoint.\n")
  cat("Visit https://genenetwork.org to browse phenotypes interactively.\n")
}
#> Total BXD published phenotype traits: 13757 
#> 
#>                                                                                                                                                                             Description
#> 1                                                                                                                         Original post publication description: Cerebellum weight [mg]
#> 2                                     Original post publication description: Central nervous system, morphology: Cerebellum weight after adjustment for covariance with brain size [mg]
#> 3                     Original post publication description: Central nervous system, morphology: Brain weight, male and female adult average, unadjusted for body weight, age, sex [mg]
#> 4                                                                                   Original post publication description: Central nervous system, morphology: Cerebellum volume [mm^3]
#> 5                                          Original post publication description: Central nervous system, morphology: Cerebellum volume, adjusted for covariance with brain size [mm^3]
#> 6                                   Original post publication description: Central nervous system, morphology: Cerebellum internal granule layer (IGL) volume without adjustment [mm^3]
#> 7  Original post publication description: Central nervous system, morphology: Internal granule layer (IGL) of the cerebellum volume, adjusted for sex, age, body and brain weight [mm3]
#> 8            Original post publication description: Phencyclidine response (7.5 mg/kg ip), locomotor response, difference in activity (PCP minus saline) between 1 hr test periods [cm]
#> 9                                               Original post publication description: Phencyclidine response (7.5 mg/kg ip), locomotor activity from 0-60 min after PCP injection [cm]
#> 10     Original post publication description: Saline control response (dose 0.9%, ip), locomotor activity from 0-60 min after injection (just prior to injection of phencyclidine) [cm]
#>       Id
#> 1  10001
#> 2  10002
#> 3  10003
#> 4  10004
#> 5  10005
#> 6  10006
#> 7  10007
#> 8  10008
#> 9  10009
#> 10 10010
```

# Integrating GeneNetwork2 Results into locusPackRat

## Create a locusPackRat Project

```
# Create a temporary project with candidate genes
project_dir <- file.path(tempdir(), "gn_demo_project")

gene_input <- data.frame(
  gene_symbol = c("Cisd2", "Pdlim5", "Manba", "Fhod3", "Myh7")
)

initPackRat(
  data = gene_input,
  mode = "gene",
  species = "mouse",
  genome = "mm39",
  project_dir = project_dir,
  force = TRUE
)
#> Initializing locusPackRat project...
#> Note: All data added to this project must use the mm39 genome build. Mixing genome builds will cause incorrect coordinate matching. Use rtracklayer::liftOver() to convert coordinates if needed.
#> Processing gene list...
#>   Matched all 5 genes to coordinates
#> Generating orthology information...
#> Saved gene data to /work/appscr/r/bgural/RtmpwHwfjY/gn_demo_project/.locusPackRat/input/genes.csv
#> Saved orthology data to /work/appscr/r/bgural/RtmpwHwfjY/gn_demo_project/.locusPackRat/input/orthology.csv
#> Created config file: /work/appscr/r/bgural/RtmpwHwfjY/gn_demo_project/.locusPackRat/config.json
#> 
#> locusPackRat project initialized successfully!
#> Mode: gene | Species: mouse | Genome: mm39
#> Processed 5 genes
```

## Gene-Level Integration

Use `link_type = "gene"` when you have per-gene summary
statistics (e.g., peak LOD score, cis/trans classification). Here we
build a summary from the GEMMA results computed above.

```
# Build a gene-level summary from the GEMMA scan
# In a real analysis, you would loop over multiple candidate genes
if (exists("eqtl_scan") && nrow(eqtl_scan) > 0) {
  peak_row <- eqtl_scan[which.max(lod)]
  gene_chr <- unique(eqtl_scan[chr == peak_row$chr, chr])

  eqtl_summary <- data.table(
    gene_symbol = gene_of_interest,
    bxd_max_lod = round(peak_row$lod, 2),
    bxd_peak_chr = as.character(peak_row$chr),
    bxd_peak_mb = round(peak_row$pos_mb, 2),
    bxd_cis_eqtl = (as.character(peak_row$chr) == as.character(cisd2_traits$Chr[1])),
    gn2_dataset = dataset_name
  )

  print(eqtl_summary)

  # Add to locusPackRat project
  addRatTable(
    data = eqtl_summary,
    table_name = "bxd_eqtl",
    abbreviation = "bxd",
    link_type = "gene",
    link_by = "gene_symbol",
    project_dir = project_dir
  )
}
#>    gene_symbol bxd_max_lod bxd_peak_chr bxd_peak_mb bxd_cis_eqtl  gn2_dataset
#>         <char>       <num>       <char>       <num>       <lgcl>       <char>
#> 1:       Cisd2         6.8            3      132.23         TRUE HC_M2_0606_P
#> Adding supplementary table to mouse mm39 project...
#> Linking data by gene_symbol...
#> Saved supplementary table to /work/appscr/r/bgural/RtmpwHwfjY/gn_demo_project/.locusPackRat/supplementary/bxd_eqtl.csv
#> Linked 1 of 1 input rows
#> Updated config file
```

## Point-Level Integration

Use `link_type = "region"` when you have position-level
QTL scan data that should be mapped to overlapping genes/regions.

```
# Use the full scan data from GEMMA -- keep significant hits only
if (exists("eqtl_scan") && nrow(eqtl_scan) > 0) {
  significant_hits <- eqtl_scan[lod > 3]
  cat("Integrating", nrow(significant_hits), "significant markers\n")

  scan_for_project <- significant_hits[, .(
    chr = as.character(chr),
    start = as.integer(pos),
    end = as.integer(pos),
    lod = round(lod, 3),
    gene_symbol = gene_symbol,
    source = source
  )]

  addRatTable(
    data = scan_for_project,
    table_name = "bxd_eqtl_scan",
    abbreviation = "bxds",
    link_type = "region",
    link_by = "chr,start,end",
    project_dir = project_dir
  )
}
#> Integrating 252 significant markers
#> Adding supplementary table to mouse mm39 project...
#> Saved supplementary table to /work/appscr/r/bgural/RtmpwHwfjY/gn_demo_project/.locusPackRat/supplementary/bxd_eqtl_scan.csv
#> Linked 252 of 252 input rows
#> Updated config file
```

## Project State After Integration

```
listPackRatTables(project_dir)
#> Found 2 supplementary table(s):
#>   - bxd_eqtl_scan: 252 rows with 6 cols, linked by 'chr,start,end'
#>   - bxd_eqtl: 1 rows with 7 cols, linked by 'gene_symbol'
#>       table_name table_abbr link_type       link_by n_rows n_cols date_added
#>           <char>     <char>    <char>        <char>  <int>  <int>     <char>
#> 1: bxd_eqtl_scan       bxds    region chr,start,end    252      6 2026-02-18
#> 2:      bxd_eqtl        bxd      gene   gene_symbol      1      7 2026-02-18
```

# Alternative: GNapi R Package

The GNapi R package by
Karl Broman provides a more structured R client for the GeneNetwork2
API. If you prefer a packaged interface:

```
# Install GNapi (https://github.com/kbroman/GNapi)
# remotes::install_github("kbroman/GNapi")
library(GNapi)

# List available species and groups
species <- list_species()
groups  <- list_groups("mouse")

# List BxD datasets
bxd_datasets <- list_datasets("BXD")

# Run GEMMA QTL scan for a specific trait
gemma_scan <- run_gemma("HC_M2_0606_P", "1428441_at")

# Find correlated traits
corr <- run_correlation("HC_M2_0606_P", "BXD", "1428441_at")
```

# Tips and Considerations

1. **API Rate Limits**: GeneNetwork2 may impose rate
   limits. Add `Sys.sleep(1)` between requests when querying
   many genes.
2. **Genome Build**: GeneNetwork2 positions are
   typically in GRCm38/mm10 for mouse data. If your locusPackRat project
   uses mm39, convert coordinates using
   `rtracklayer::liftOver()` before integration (see the
   liftOver section in the main workflow vignette).
3. **Dataset Selection**: Choose tissue-appropriate
   datasets. For a cardiac study, prioritize heart expression datasets over
   brain or liver.
4. **Cis vs Trans**: A cis-eQTL (peak near the gene
   itself) is generally stronger evidence for candidacy than a
   trans-eQTL.
5. **Cross-Validation**: Compare GeneNetwork2 eQTL
   results with your own study-specific eQTL data and Open Targets QTL data
   for convergent evidence.

# Session Info

```
sessionInfo()
#> R version 4.5.2 (2025-10-31)
#> Platform: x86_64-conda-linux-gnu
#> Running under: Red Hat Enterprise Linux 9.7 (Plow)
#> 
#> Matrix products: default
#> BLAS/LAPACK: /nas/longleaf/home/bgural/mambaforge/envs/packrat_dev/lib/libopenblasp-r0.3.30.so;  LAPACK version 3.12.0
#> 
#> locale:
#>  [1] LC_CTYPE=en_US.UTF-8       LC_NUMERIC=C              
#>  [3] LC_TIME=en_US.UTF-8        LC_COLLATE=en_US.UTF-8    
#>  [5] LC_MONETARY=en_US.UTF-8    LC_MESSAGES=en_US.UTF-8   
#>  [7] LC_PAPER=en_US.UTF-8       LC_NAME=C                 
#>  [9] LC_ADDRESS=C               LC_TELEPHONE=C            
#> [11] LC_MEASUREMENT=en_US.UTF-8 LC_IDENTIFICATION=C       
#> 
#> time zone: America/New_York
#> tzcode source: system (glibc)
#> 
#> attached base packages:
#> [1] stats     graphics  grDevices utils     datasets  methods   base     
#> 
#> other attached packages:
#> [1] jsonlite_2.0.0     httr_1.4.8         data.table_1.17.8  locusPackRat_0.6.2
#> 
#> loaded via a namespace (and not attached):
#>  [1] SummarizedExperiment_1.36.0 gtable_0.3.6               
#>  [3] rjson_0.2.23                xfun_0.56                  
#>  [5] bslib_0.10.0                ggplot2_4.0.2              
#>  [7] plyranges_1.26.0            rhdf5_2.50.2               
#>  [9] Biobase_2.66.0              lattice_0.22-9             
#> [11] rhdf5filters_1.18.1         bitops_1.0-9               
#> [13] vctrs_0.7.1                 tools_4.5.2                
#> [15] generics_0.1.4              yulab.utils_0.2.4          
#> [17] parallel_4.5.2              stats4_4.5.2               
#> [19] curl_7.0.0                  tibble_3.3.1               
#> [21] pkgconfig_2.0.3             Matrix_1.7-4               
#> [23] ggplotify_0.1.3             plotgardener_1.12.0        
#> [25] RColorBrewer_1.1-3          S7_0.2.1                   
#> [27] S4Vectors_0.48.0            lifecycle_1.0.5            
#> [29] GenomeInfoDbData_1.2.13     compiler_4.5.2             
#> [31] farver_2.1.2                Rsamtools_2.22.0           
#> [33] Biostrings_2.74.1           codetools_0.2-20           
#> [35] GenomeInfoDb_1.42.3         htmltools_0.5.9            
#> [37] sass_0.4.10                 RCurl_1.98-1.17            
#> [39] yaml_2.3.12                 pillar_1.11.1              
#> [41] crayon_1.5.3                jquerylib_0.1.4            
#> [43] BiocParallel_1.40.2         cachem_1.1.0               
#> [45] DelayedArray_0.32.0         abind_1.4-8                
#> [47] tidyselect_1.2.1            zip_2.3.3                  
#> [49] digest_0.6.39               stringi_1.8.7              
#> [51] purrr_1.2.1                 restfulr_0.0.16            
#> [53] dplyr_1.2.0                 fastmap_1.2.0              
#> [55] grid_4.5.2                  SparseArray_1.6.2          
#> [57] cli_3.6.5                   magrittr_2.0.4             
#> [59] S4Arrays_1.6.0              XML_3.99-0.20              
#> [61] withr_3.0.2                 scales_1.4.0               
#> [63] UCSC.utils_1.2.0            rappdirs_0.3.4             
#> [65] rmarkdown_2.30              XVector_0.46.0             
#> [67] matrixStats_1.5.0           otel_0.2.0                 
#> [69] openxlsx_4.2.8.1            evaluate_1.0.5             
#> [71] knitr_1.51                  BiocIO_1.16.0              
#> [73] GenomicRanges_1.58.0        IRanges_2.40.1             
#> [75] rtracklayer_1.66.0          gridGraphics_0.5-1         
#> [77] rlang_1.1.7                 Rcpp_1.1.1                 
#> [79] glue_1.8.0                  BiocGenerics_0.56.0        
#> [81] strawr_0.0.92               Rhdf5lib_1.28.0            
#> [83] R6_2.6.1                    MatrixGenerics_1.18.1      
#> [85] GenomicAlignments_1.42.0    fs_1.6.6                   
#> [87] zlibbioc_1.52.0
```
