# Supplementary material for: LocusPackRat: an R package to support prioritizing candidate genes from large GWAS intervals with standardized evidence aggregation
Source: G3 (Bethesda). 2026 Mar 28;16(6):jkag081. doi: 10.1093/g3journal/jkag081 (PMC13232493; doi:10.1093/g3journal/jkag081)
Supplement: jkag081_Supplementary_Data [file jkag081_supplementary_data.zip › Supplementary_File_4_G3-2026-406637.html]

Single-Cell RNA-seq Integration for Cell-Type-Specific Candidate Evaluation


# Single-Cell RNA-seq Integration for Cell-Type-Specific Candidate Evaluation

#### Brian Gural, Todd Kimball, Anh Luu, Christoph D. Rau

#### 2026-02-18

- Overview
- Prerequisites
- Step 1: Create a
  locusPackRat Project
- Step 2: Load a
  Reference scRNA-seq Dataset
- Step 3: Score
  Candidate Genes with AddModuleScore
- Step 4: Visualize
  Module Scores by Cell Type
  - UMAP
    Visualization
  - Violin Plot by Cell Type
  - Individual Gene Expression
- Step 5: Quantify
  Cell-Type Specificity
- Step 6: Integrate
  Results into locusPackRat
  - Project State After
    Integration
- Tips and Considerations
- Session Info

# Overview

Bulk-tissue transcriptomic data, which form a common evidence layer
in GWAS candidate gene prioritization, can obscure effects that are
specific to particular cell types. A gene that appears broadly expressed
in bulk tissue may in fact be restricted to a small cell population, and
conversely, a gene with moderate bulk expression may be highly enriched
in the disease-relevant cell type.

This vignette demonstrates how to use single-cell RNA-seq (scRNA-seq)
data to evaluate candidate genes identified by `locusPackRat`
for cell-type-specific expression. Specifically, we show how to:

1. Load a reference scRNA-seq dataset
2. Score candidate gene sets using Seurat’s
   `AddModuleScore()`
3. Visualize module scores across cell types
4. Use the results to refine candidate gene prioritization

A bundled mini Seurat object (200 cells, 4 cardiac cell types) is
used here for demonstration. For real analyses, substitute your own
scRNA-seq reference (see the Tabula Muris example at the end of this
vignette).

# Prerequisites

```
# Core packages
library(locusPackRat)
library(data.table)

# scRNA-seq analysis
library(Seurat)     # install from CRAN: install.packages("Seurat")
#> Loading required package: SeuratObject
#> Loading required package: sp
#> 
#> Attaching package: 'SeuratObject'
#> The following objects are masked from 'package:base':
#> 
#>     intersect, t
library(ggplot2)
```

# Step 1: Create a locusPackRat Project

First, we create a temporary locusPackRat project with candidate
genes from a cardiac hypertrophy study. These five genes will be
evaluated for cell-type specificity.

```
# Define candidate genes from a cardiac QTL study
cardiac_candidates <- c("Cisd2", "Pdlim5", "Manba", "Fhod3", "Myh7")

# Create a temporary project directory
project_dir <- file.path(tempdir(), "sc_demo_project")

# Initialize a locusPackRat project with these genes
gene_input <- data.frame(gene_symbol = cardiac_candidates)
initPackRat(
  data = gene_input,
  mode = "gene",
  species = "mouse",
  genome = "mm39",
  project_dir = project_dir,
  force = TRUE
)
#> Initializing locusPackRat project...
#> Note: All data added to this project must use the mm39 genome build. Mixing genome builds will cause incorrect coordinate matching. Use rtracklayer::liftOver() to convert coordinates if needed.
#> Processing gene list...
#>   Matched all 5 genes to coordinates
#> Generating orthology information...
#> Saved gene data to /work/appscr/r/bgural/RtmpwHwfjY/sc_demo_project/.locusPackRat/input/genes.csv
#> Saved orthology data to /work/appscr/r/bgural/RtmpwHwfjY/sc_demo_project/.locusPackRat/input/orthology.csv
#> Created config file: /work/appscr/r/bgural/RtmpwHwfjY/sc_demo_project/.locusPackRat/config.json
#> 
#> locusPackRat project initialized successfully!
#> Mode: gene | Species: mouse | Genome: mm39
#> Processed 5 genes

# Show the initialized gene table
master_genes <- fread(file.path(project_dir, ".locusPackRat", "input", "genes.csv"))
print(master_genes[, .(gene_symbol, chr, start, end)])
#>    gene_symbol   chr     start       end
#>         <char> <int>     <int>     <int>
#> 1:       Cisd2     3 135112173 135129686
#> 2:       Fhod3    18  24841680  25266558
#> 3:       Manba     3 135191372 135277165
#> 4:        Myh7    14  55208141  55232083
#> 5:      Pdlim5     3 141945351 142101454
```

# Step 2: Load a Reference scRNA-seq Dataset

We load the bundled mini Seurat object, which contains 200 cells
across four cardiac cell types (cardiomyocyte, fibroblast, endothelial,
immune) with pre-computed PCA and UMAP embeddings.

```
# Load the bundled mini Seurat object
seurat_path <- system.file("extdata", "mini_heart_seurat.rds",
                           package = "locusPackRat")
# Fallback for devtools/development builds where inst/ isn't installed yet
if (!nzchar(seurat_path)) {
  seurat_path <- file.path("..", "inst", "extdata", "mini_heart_seurat.rds")
}

if (!file.exists(seurat_path)) {
  message("mini_heart_seurat.rds not found -- skipping remaining chunks.")
  knitr::opts_chunk$set(eval = FALSE)
} else {
  heart_seurat <- readRDS(seurat_path)

  # Inspect the object
  heart_seurat

  # Cell type composition
  table(heart_seurat$cell_type)
}
#> 
#> cardiomyocyte   endothelial    fibroblast        immune 
#>            50            50            50            50
```

For a real analysis, you would load a full scRNA-seq reference such
as Tabula Muris. The code below is provided as a reference but not
executed:

```
# Download Tabula Muris heart data (10X Genomics)
# Data available from: https://tabula-muris.ds.czbiohub.org/
heart_data <- Read10X(data.dir = "path/to/tabula_muris/Heart-10X_P7_4/")
heart_seurat <- CreateSeuratObject(counts = heart_data, project = "TabulaMuris_Heart",
                                   min.cells = 3, min.features = 200)
heart_seurat <- NormalizeData(heart_seurat)
heart_seurat <- FindVariableFeatures(heart_seurat, nfeatures = 2000)
heart_seurat <- ScaleData(heart_seurat)
heart_seurat <- RunPCA(heart_seurat)
heart_seurat <- FindNeighbors(heart_seurat, dims = 1:20)
heart_seurat <- FindClusters(heart_seurat, resolution = 0.5)
heart_seurat <- RunUMAP(heart_seurat, dims = 1:20)
```

# Step 3: Score Candidate Genes with AddModuleScore

Seurat’s `AddModuleScore()` calculates per-cell enrichment
scores for a set of genes, controlling for expression level and gene set
size. This allows us to ask: “Are our candidate genes collectively
enriched in specific cell types?”

```
# Only score genes that are present in the dataset
candidates_in_data <- intersect(cardiac_candidates, rownames(heart_seurat))
cat("Candidates found in dataset:", paste(candidates_in_data, collapse = ", "), "\n")
#> Candidates found in dataset: Cisd2, Pdlim5, Manba, Fhod3, Myh7

# Define gene lists for module scoring
candidate_list <- list(cardiac_candidates = candidates_in_data)

# Calculate module scores
# For small gene panels (e.g., bundled demo data), reduce ctrl and nbin
# so that control gene sampling does not exceed available genes per bin
n_features <- nrow(heart_seurat)
use_ctrl <- if (n_features < 200) 5 else 100
use_nbin <- if (n_features < 200) 5 else 24
heart_seurat <- AddModuleScore(
  object = heart_seurat,
  features = candidate_list,
  ctrl = use_ctrl,
  nbin = use_nbin,
  name = "candidate_score"
)

# The score is added as a metadata column: "candidate_score1"
# Higher scores indicate stronger collective expression of the candidate genes
summary(heart_seurat$candidate_score1)
#>    Min. 1st Qu.  Median    Mean 3rd Qu.    Max. 
#> -3.4598 -1.9638 -1.0709 -1.0066 -0.1324  1.8566
```

# Step 4: Visualize Module Scores by Cell Type

## UMAP Visualization

The module score projected onto the UMAP embedding reveals which cell
populations show enriched expression of the candidate gene set.

```
FeaturePlot(
  heart_seurat,
  features = "candidate_score1",
  cols = c("lightgrey", "blue", "red"),
  pt.size = 1.5
) +
  ggtitle("Candidate Gene Module Score") +
  theme(plot.title = element_text(hjust = 0.5))
```

UMAP colored by candidate gene module score.
Warmer colors indicate higher collective expression.

## Violin Plot by Cell Type

A violin plot directly compares the distribution of module scores
across cell types.

```
VlnPlot(
  heart_seurat,
  features = "candidate_score1",
  group.by = "cell_type",
  pt.size = 0
) +
  ggtitle("Candidate Gene Enrichment by Cell Type") +
  theme(axis.text.x = element_text(angle = 45, hjust = 1))
```

Module score distribution by cell type.
Cardiomyocytes show the highest enrichment.

## Individual Gene Expression

Examining expression of individual candidate genes across cell types
identifies which candidates drive the module score.

```
# Use only candidates present in the data
genes_to_plot <- intersect(cardiac_candidates, rownames(heart_seurat))

DotPlot(
  heart_seurat,
  features = genes_to_plot,
  group.by = "cell_type"
) +
  RotatedAxis() +
  ggtitle("Individual Candidate Gene Expression by Cell Type")
#> Warning: Scaling data with a low number of groups may produce misleading
#> results
```

Dot plot of individual candidate genes by cell
type. Size = percent expressing; color = mean expression.

# Step 5: Quantify Cell-Type Specificity

Calculate summary statistics for candidate gene expression across
cell types to produce a quantitative ranking.

```
# Extract expression matrix for candidate genes
# Use layer= for Seurat v5 compatibility, fall back to slot= for v4
expr_matrix <- tryCatch(
  GetAssayData(heart_seurat, layer = "data"),
  error = function(e) GetAssayData(heart_seurat, slot = "data")
)
candidates_in_data <- intersect(cardiac_candidates, rownames(expr_matrix))

if (length(candidates_in_data) > 0) {
  # Calculate mean expression per cell type per gene
  cell_type_expr <- data.table(
    cell_type = heart_seurat$cell_type,
    as.data.table(t(as.matrix(expr_matrix[candidates_in_data, , drop = FALSE])))
  )

  # Aggregate by cell type
  mean_expr <- cell_type_expr[, lapply(.SD, mean), by = cell_type,
                               .SDcols = candidates_in_data]

  # Calculate percentage of cells expressing each gene (> 0) per cell type
  pct_expr <- cell_type_expr[, lapply(.SD, function(x) mean(x > 0) * 100),
                              by = cell_type, .SDcols = candidates_in_data]

  cat("Mean expression by cell type:\n")
  print(mean_expr)

  cat("\nPercent cells expressing (> 0) by cell type:\n")
  print(pct_expr)
}
#> Mean expression by cell type:
#>        cell_type    Cisd2   Pdlim5    Manba     Fhod3      Myh7
#>           <char>    <num>    <num>    <num>     <num>     <num>
#> 1: cardiomyocyte 5.309071 1.237711 2.930222 5.8097861 5.7916169
#> 2:    fibroblast 4.482631 5.845352 1.124158 1.4648019 1.0245894
#> 3:   endothelial 4.873538 1.442376 1.151545 1.7073730 0.7211630
#> 4:        immune 2.944514 1.202297 5.340340 0.9442931 0.7846961
#> 
#> Percent cells expressing (> 0) by cell type:
#>        cell_type Cisd2 Pdlim5 Manba Fhod3  Myh7
#>           <char> <num>  <num> <num> <num> <num>
#> 1: cardiomyocyte    98     30    66   100   100
#> 2:    fibroblast    92    100    26    34    24
#> 3:   endothelial    96     32    26    38    16
#> 4:        immune    64     28    98    22    18
```

# Step 6: Integrate Results into locusPackRat

The cell-type specificity results can be formatted and added to a
locusPackRat project as an additional evidence layer.

```
# Build a summary table of cell-type expression for each candidate
cell_type_names <- sort(unique(heart_seurat$cell_type))

specificity_summary <- data.table(
  gene_symbol = candidates_in_data,
  cardiomyocyte_expr = as.numeric(mean_expr[cell_type == "cardiomyocyte",
                                             ..candidates_in_data]),
  fibroblast_expr = as.numeric(mean_expr[cell_type == "fibroblast",
                                          ..candidates_in_data]),
  endothelial_expr = as.numeric(mean_expr[cell_type == "endothelial",
                                           ..candidates_in_data]),
  immune_expr = as.numeric(mean_expr[cell_type == "immune",
                                      ..candidates_in_data])
)

# Calculate a simple specificity index:
# ratio of cardiomyocyte expression to mean of all other cell types
specificity_summary[, cardiomyocyte_specificity :=
  cardiomyocyte_expr / rowMeans(.SD),
  .SDcols = c("fibroblast_expr", "endothelial_expr", "immune_expr")]

print(specificity_summary)
#>    gene_symbol cardiomyocyte_expr fibroblast_expr endothelial_expr immune_expr
#>         <char>              <num>           <num>            <num>       <num>
#> 1:       Cisd2           5.309071        4.482631         4.873538   2.9445143
#> 2:      Pdlim5           1.237711        5.845352         1.442376   1.2022968
#> 3:       Manba           2.930222        1.124158         1.151545   5.3403399
#> 4:       Fhod3           5.809786        1.464802         1.707373   0.9442931
#> 5:        Myh7           5.791617        1.024589         0.721163   0.7846961
#>    cardiomyocyte_specificity
#>                        <num>
#> 1:                 1.2948236
#> 2:                 0.4373525
#> 3:                 1.1542299
#> 4:                 4.2340566
#> 5:                 6.8663130

# Add to locusPackRat project
addRatTable(
  data = specificity_summary,
  table_name = "sc_cell_type_expression",
  abbreviation = "sc",
  link_type = "gene",
  link_by = "gene_symbol",
  project_dir = project_dir
)
#> Adding supplementary table to mouse mm39 project...
#> Linking data by gene_symbol...
#> Saved supplementary table to /work/appscr/r/bgural/RtmpwHwfjY/sc_demo_project/.locusPackRat/supplementary/sc_cell_type_expression.csv
#> Linked 5 of 5 input rows
#> Updated config file
```

## Project State After Integration

```
# Show all tables in the project
listPackRatTables(project_dir)
#> Found 1 supplementary table(s):
#>   - sc_cell_type_expression: 5 rows with 7 cols, linked by 'gene_symbol'
#>                 table_name table_abbr link_type     link_by n_rows n_cols
#>                     <char>     <char>    <char>      <char>  <int>  <int>
#> 1: sc_cell_type_expression         sc      gene gene_symbol      5      7
#>    date_added
#>        <char>
#> 1: 2026-02-18
```

The single-cell evidence layer is now stored alongside any other
annotations in the project. It can be exported via
`buildExcel()` or used for downstream prioritization.

# Tips and Considerations

1. **Reference Dataset Selection**: Choose a scRNA-seq
   dataset from the tissue most relevant to your phenotype. Tabula Muris
   provides broad tissue coverage; tissue-specific atlases (e.g., the Human
   Cell Atlas) may offer finer resolution.
2. **Species Matching**: If your locusPackRat project
   uses mouse gene symbols, ensure the scRNA-seq reference also uses mouse
   symbols. For cross-species comparisons, use the orthology table
   generated by `initPackRat()`.
3. **Module Score Interpretation**:
   `AddModuleScore()` returns relative scores that control for
   average expression level. A high score means the gene set is more
   expressed than expected given the cells’ overall transcriptional
   activity. Scores are most useful for comparing across cell types within
   the same dataset, not across datasets.
4. **Statistical Testing**: For formal statistical
   comparisons of module scores between cell types, consider using a
   Wilcoxon rank-sum test or permutation testing rather than relying solely
   on visual inspection.
5. **Scaling to Many Loci**: For studies with many
   candidate loci, you can create separate module scores for each locus and
   compare cell-type enrichment patterns across loci to identify shared or
   locus-specific cell type involvement.
6. **Single-Cell QTL Data**: As single-cell eQTL
   (sceQTL) datasets grow, consider querying these through
   `queryOpenTargetsQTL()` with
   `study_types = c("sceqtl", "scpqtl")` for variant-level
   cell-type-specific evidence that complements the expression-based
   approach shown here.

# Session Info

```
sessionInfo()
#> R version 4.5.2 (2025-10-31)
#> Platform: x86_64-conda-linux-gnu
#> Running under: Red Hat Enterprise Linux 9.7 (Plow)
#> 
#> Matrix products: default
#> BLAS/LAPACK: /nas/longleaf/home/bgural/mambaforge/envs/packrat_dev/lib/libopenblasp-r0.3.30.so;  LAPACK version 3.12.0
#> 
#> locale:
#>  [1] LC_CTYPE=en_US.UTF-8       LC_NUMERIC=C              
#>  [3] LC_TIME=en_US.UTF-8        LC_COLLATE=en_US.UTF-8    
#>  [5] LC_MONETARY=en_US.UTF-8    LC_MESSAGES=en_US.UTF-8   
#>  [7] LC_PAPER=en_US.UTF-8       LC_NAME=C                 
#>  [9] LC_ADDRESS=C               LC_TELEPHONE=C            
#> [11] LC_MEASUREMENT=en_US.UTF-8 LC_IDENTIFICATION=C       
#> 
#> time zone: America/New_York
#> tzcode source: system (glibc)
#> 
#> attached base packages:
#> [1] stats     graphics  grDevices utils     datasets  methods   base     
#> 
#> other attached packages:
#> [1] ggplot2_4.0.2      Seurat_5.4.0       SeuratObject_5.3.0 sp_2.2-1          
#> [5] jsonlite_2.0.0     httr_1.4.8         data.table_1.17.8  locusPackRat_0.6.2
#> 
#> loaded via a namespace (and not attached):
#>   [1] RcppAnnoy_0.0.23                         
#>   [2] splines_4.5.2                            
#>   [3] later_1.4.6                              
#>   [4] BiocIO_1.16.0                            
#>   [5] bitops_1.0-9                             
#>   [6] ggplotify_0.1.3                          
#>   [7] tibble_3.3.1                             
#>   [8] polyclip_1.10-7                          
#>   [9] XML_3.99-0.20                            
#>  [10] fastDummies_1.7.5                        
#>  [11] lifecycle_1.0.5                          
#>  [12] globals_0.19.0                           
#>  [13] lattice_0.22-9                           
#>  [14] MASS_7.3-65                              
#>  [15] magrittr_2.0.4                           
#>  [16] openxlsx_4.2.8.1                         
#>  [17] plotly_4.12.0                            
#>  [18] sass_0.4.10                              
#>  [19] rmarkdown_2.30                           
#>  [20] jquerylib_0.1.4                          
#>  [21] yaml_2.3.12                              
#>  [22] httpuv_1.6.16                            
#>  [23] otel_0.2.0                               
#>  [24] sctransform_0.4.3                        
#>  [25] spam_2.11-3                              
#>  [26] zip_2.3.3                                
#>  [27] spatstat.sparse_3.1-0                    
#>  [28] plotgardener_1.12.0                      
#>  [29] reticulate_1.45.0                        
#>  [30] cowplot_1.2.0                            
#>  [31] pbapply_1.7-4                            
#>  [32] DBI_1.2.3                                
#>  [33] RColorBrewer_1.1-3                       
#>  [34] abind_1.4-8                              
#>  [35] zlibbioc_1.52.0                          
#>  [36] Rtsne_0.17                               
#>  [37] GenomicRanges_1.58.0                     
#>  [38] purrr_1.2.1                              
#>  [39] BiocGenerics_0.56.0                      
#>  [40] RCurl_1.98-1.17                          
#>  [41] yulab.utils_0.2.4                        
#>  [42] rappdirs_0.3.4                           
#>  [43] GenomeInfoDbData_1.2.13                  
#>  [44] IRanges_2.40.1                           
#>  [45] S4Vectors_0.48.0                         
#>  [46] ggrepel_0.9.6                            
#>  [47] irlba_2.3.7                              
#>  [48] spatstat.utils_3.2-1                     
#>  [49] listenv_0.10.0                           
#>  [50] goftest_1.2-3                            
#>  [51] RSpectra_0.16-2                          
#>  [52] spatstat.random_3.4-4                    
#>  [53] TxDb.Mmusculus.UCSC.mm39.knownGene_3.20.0
#>  [54] fitdistrplus_1.2-6                       
#>  [55] parallelly_1.46.1                        
#>  [56] codetools_0.2-20                         
#>  [57] DelayedArray_0.32.0                      
#>  [58] tidyselect_1.2.1                         
#>  [59] UCSC.utils_1.2.0                         
#>  [60] farver_2.1.2                             
#>  [61] spatstat.explore_3.7-0                   
#>  [62] matrixStats_1.5.0                        
#>  [63] stats4_4.5.2                             
#>  [64] GenomicAlignments_1.42.0                 
#>  [65] progressr_0.18.0                         
#>  [66] ggridges_0.5.7                           
#>  [67] survival_3.8-6                           
#>  [68] tools_4.5.2                              
#>  [69] strawr_0.0.92                            
#>  [70] ica_1.0-3                                
#>  [71] Rcpp_1.1.1                               
#>  [72] glue_1.8.0                               
#>  [73] gridExtra_2.3                            
#>  [74] SparseArray_1.6.2                        
#>  [75] xfun_0.56                                
#>  [76] MatrixGenerics_1.18.1                    
#>  [77] GenomeInfoDb_1.42.3                      
#>  [78] dplyr_1.2.0                              
#>  [79] withr_3.0.2                              
#>  [80] fastmap_1.2.0                            
#>  [81] rhdf5filters_1.18.1                      
#>  [82] digest_0.6.39                            
#>  [83] R6_2.6.1                                 
#>  [84] mime_0.13                                
#>  [85] gridGraphics_0.5-1                       
#>  [86] scattermore_1.2                          
#>  [87] tensor_1.5.1                             
#>  [88] spatstat.data_3.1-9                      
#>  [89] RSQLite_2.4.4                            
#>  [90] tidyr_1.3.2                              
#>  [91] generics_0.1.4                           
#>  [92] rtracklayer_1.66.0                       
#>  [93] htmlwidgets_1.6.4                        
#>  [94] S4Arrays_1.6.0                           
#>  [95] org.Mm.eg.db_3.20.0                      
#>  [96] uwot_0.2.4                               
#>  [97] pkgconfig_2.0.3                          
#>  [98] gtable_0.3.6                             
#>  [99] blob_1.2.4                               
#> [100] lmtest_0.9-40                            
#> [101] S7_0.2.1                                 
#> [102] XVector_0.46.0                           
#> [103] htmltools_0.5.9                          
#> [104] dotCall64_1.2                            
#> [105] plyranges_1.26.0                         
#> [106] scales_1.4.0                             
#> [107] Biobase_2.66.0                           
#> [108] TxDb.Hsapiens.UCSC.hg38.knownGene_3.20.0 
#> [109] png_0.1-8                                
#> [110] spatstat.univar_3.1-6                    
#> [111] knitr_1.51                               
#> [112] reshape2_1.4.5                           
#> [113] rjson_0.2.23                             
#> [114] nlme_3.1-168                             
#> [115] curl_7.0.0                               
#> [116] org.Hs.eg.db_3.20.0                      
#> [117] cachem_1.1.0                             
#> [118] zoo_1.8-15                               
#> [119] rhdf5_2.50.2                             
#> [120] stringr_1.6.0                            
#> [121] KernSmooth_2.23-26                       
#> [122] parallel_4.5.2                           
#> [123] miniUI_0.1.2                             
#> [124] AnnotationDbi_1.68.0                     
#> [125] restfulr_0.0.16                          
#> [126] pillar_1.11.1                            
#> [127] grid_4.5.2                               
#> [128] vctrs_0.7.1                              
#> [129] RANN_2.6.2                               
#> [130] promises_1.5.0                           
#> [131] xtable_1.8-4                             
#> [132] cluster_2.1.8.2                          
#> [133] evaluate_1.0.5                           
#> [134] GenomicFeatures_1.58.0                   
#> [135] cli_3.6.5                                
#> [136] compiler_4.5.2                           
#> [137] Rsamtools_2.22.0                         
#> [138] rlang_1.1.7                              
#> [139] crayon_1.5.3                             
#> [140] future.apply_1.20.1                      
#> [141] labeling_0.4.3                           
#> [142] plyr_1.8.9                               
#> [143] fs_1.6.6                                 
#> [144] stringi_1.8.7                            
#> [145] deldir_2.0-4                             
#> [146] viridisLite_0.4.3                        
#> [147] BiocParallel_1.40.2                      
#> [148] Biostrings_2.74.1                        
#> [149] lazyeval_0.2.2                           
#> [150] spatstat.geom_3.7-0                      
#> [151] Matrix_1.7-4                             
#> [152] RcppHNSW_0.6.0                           
#> [153] patchwork_1.3.2                          
#> [154] bit64_4.6.0-1                            
#> [155] future_1.69.0                            
#> [156] Rhdf5lib_1.28.0                          
#> [157] KEGGREST_1.46.0                          
#> [158] shiny_1.12.1                             
#> [159] SummarizedExperiment_1.36.0              
#> [160] ROCR_1.0-12                              
#> [161] igraph_2.1.4                             
#> [162] memoise_2.0.1                            
#> [163] bslib_0.10.0                             
#> [164] bit_4.6.0
```
