# Supplementary figures and images for: LocusPackRat: an R package to support prioritizing candidate genes from large GWAS intervals with standardized evidence aggregation
Source: G3 (Bethesda). 2026 Mar 28;16(6):jkag081. doi: 10.1093/g3journal/jkag081 (PMC13232493; doi:10.1093/g3journal/jkag081)

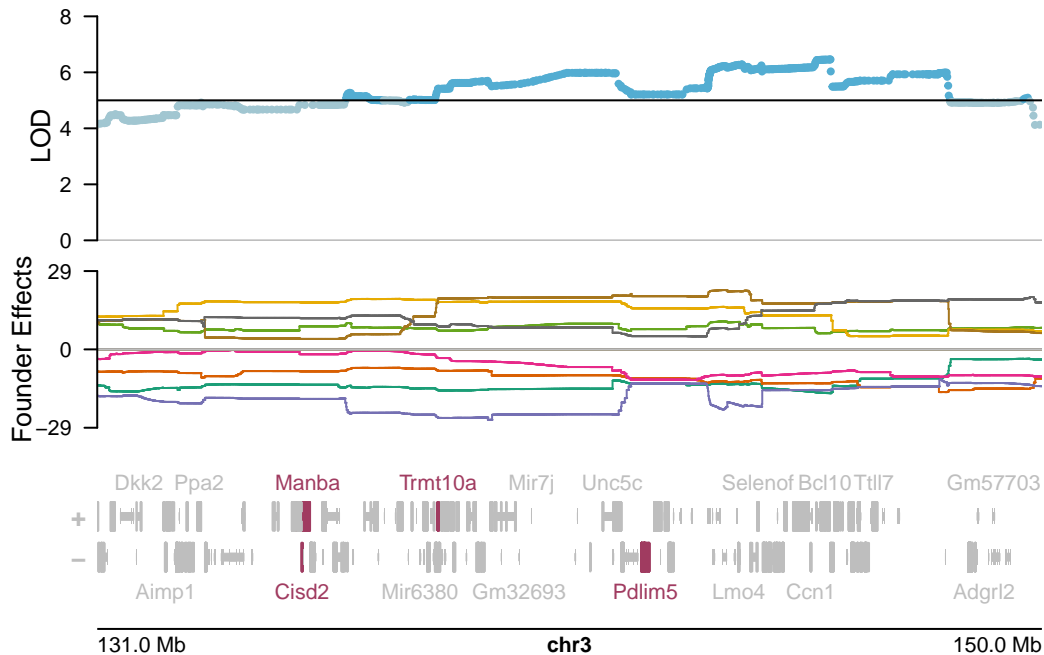

Supplement: jkag081_Supplementary_Data [file jkag081_supplementary_data.zip › EF21_chr3_locus_zoom.pdf]
